# Artificial Internalizing Receptors for Targeted Degradation of Extracellular Proteins

**DOI:** 10.1002/advs.202509083

**Published:** 2025-07-10

**Authors:** Ane B. Søgaard, Rikke Fabech Hansson, Mikkel Høj Nielsen, Alexander N. Zelikin

**Affiliations:** ^1^ Department of Chemistry Aarhus University Aarhus C 8000 Denmark; ^2^ iNano Interdisciplinary Nanoscience Center Aarhus University Aarhus C 8000 Denmark

**Keywords:** artificial receptors, cell engineering, cell surface, targeted protein degradation

## Abstract

Artificial internalizing receptors are developed, and it is demonstrated that these small organic molecules are powerful agents for capturing and removing the cognate protein of interest from the extracellular space. Toward the overall goal, a range of candidate receptor molecules is synthesized, and the leads are selected based on the highest efficacy of protein capture and internalization by the receptor‐engineered cells. Receptor performance is validated in model T lymphoblasts, donor‐derived peripheral blood mononuclear cells, and hepatocytes. The capture and depletion are achieved over successive additions of the extracellular target protein.  In contrast to pan‐IgG degraders, artificial receptors depleted the one, cognate immunoglobulin, leaving the pool of non‐cognate proteins un‐altered. In an in vitro model system, mimicking hepatic removal of the target protein, receptor‐engineered scavenger hepatocytes captured the protein of interest and prevented its association with the cognate receptor on the responder lymphoblasts.

## Graphical abstract

Small organic molecules that are anchored into the lipid bilayer of a mammalian cell and have an exofacial recognition ligand can act as artificial internalizing receptors. These receptors are an efficacious tool to selectively capture and internalize cognate antibodies from the extracellular environment in a protein‐specific manner.

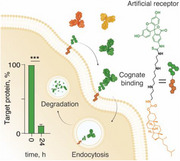

## 1 Introduction

Receptors are a broad class of molecules that comprise the biomolecular fingerprint of the cell and thus define the interaction of the living cell with the outer surroundings. By structure, receptors are most typically large, often multi‐domain proteins but this class of molecules also includes lipids such as gangliosides.^[^ ^1^, ^2^ ^]^ The predominant function of receptors is recognition, an essential phenomenon that mediates cellular functions such as adhesion, cohesion, immune surveillance, etc. Recognition is often associated with transmembrane signaling, whereby a binding event at the receptor, at the cell surface, leads to an intracellular response.^[^ ^1^, ^2^ ^]^ This binding event can also lead to the internalization of the receptor and the associated ligand.^[^ ^1^, ^3^ ^]^ Historically, the latter phenomenon has been widely used in the context of targeted drug delivery.^[^ ^4^ ^]^ In recent years, receptor mediated endocytosis re‐emerged in the focus of biomedical sciences as the key player in performing targeted capture and degradation of extracellular proteins.^[^ ^5^, ^6^ ^]^

Targeted protein degradation (TPD) is a new, rapidly maturing modality in medicinal chemistry that aims to eliminate a disease‐related target protein using the cellular protein degradation machinery.^[^ ^6^, ^7^ ^]^ For the extracellular proteins, this is typically accomplished using divalent, bi‐functional soluble factors based on proteins,^[^ ^8^, ^9^, ^10^ ^]^ small molecules,^[^ ^11^ ^]^ or a combination thereof,^[^ ^12^ ^]^ with the overall aim to bridge the target protein to a natural internalizing receptor that guides the captured protein along the lysosomal degradation pathway. In an early example, Banik et al. designed the so‐called “lysosome targeting chimeras” (LYTACs), which featured an antibody to bind the protein of interest and a ligand that binds the cation‐independent mannose‐6‐phosphate receptor.^[^ ^5^ ^]^ Other natural receptors used for internalization of the captured proteins include the liver‐specific asialglycoprotein receptor,^[^ ^13^ ^]^ cytokine‐specific receptors,^[^ ^10^ ^]^ integrins,^[^ ^14^ ^]^ the neonatal fragment crystallizable receptors (FcRn),^[^ ^8^ ^]^ and even glucose transporter proteins.^[^ ^12^ ^]^

**Figure 1:**
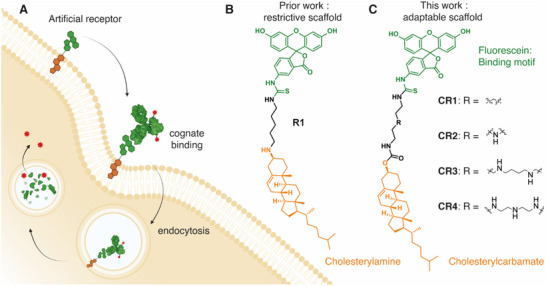
A) Schematic illustration of the artificial receptor embedded in the plasma membrane of a mammalian cell, receptor association with the cognate protein, and the ensuing internalization of the protein along the endosomal/lysosomal trafficking pathway; B) the structure of previously‐published artificial internalizing receptor design based on cholesterylamine anchor; C) the general structure of the artificial internalizing receptors engineered in this work whereby cholesterylcarbamate acts as the bilayer anchor, fluorescein acts as the exofacial recognition ligand for the cognate antibody, and the polyamine linker is selected to optimize the performance of the artificial receptors. Molecules and cellular (sub)compartments are not drawn to scale.

The use of natural receptors for targeted protein degradation is elegant and powerful,^[^ ^6^, ^10^, ^11^, ^12^, ^14^ ^]^ but may not be without limitations. Specifically, i) the expression level of the endogenous internalizing receptors can be highly variable; ii) enforced internalization of the endogenous receptors can lead to a loss of the natural function associated with this receptor¸ iii) the pharmacokinetics of the divalent capture agent should likely be aligned to that of the target protein. To address these shortcomings, in this work, we develop targeted capture and degradation of extracellular proteins via chemical engineering of the cell surface^[^ ^15^, ^16^, ^17^ ^]^ and specifically, through the design of artificial internalizing receptors (**Figure** **1**A). These small molecules integrate into the lipid bilayer of the cell, and the receptor content in the bilayer is controlled via the receptor dosing during the chemical cell engineering protocol. The molecules are bio‐orthogonal and perform their function within the otherwise un‐altered cell, they persist in the cell for days, and can continuously deplete the target extracellular protein. We believe that these data demonstrate that artificial internalizing receptors are a powerful alternative to their natural counterpart in the arsenal of tools used to achieve targeted protein degradation.

**Figure 2:**
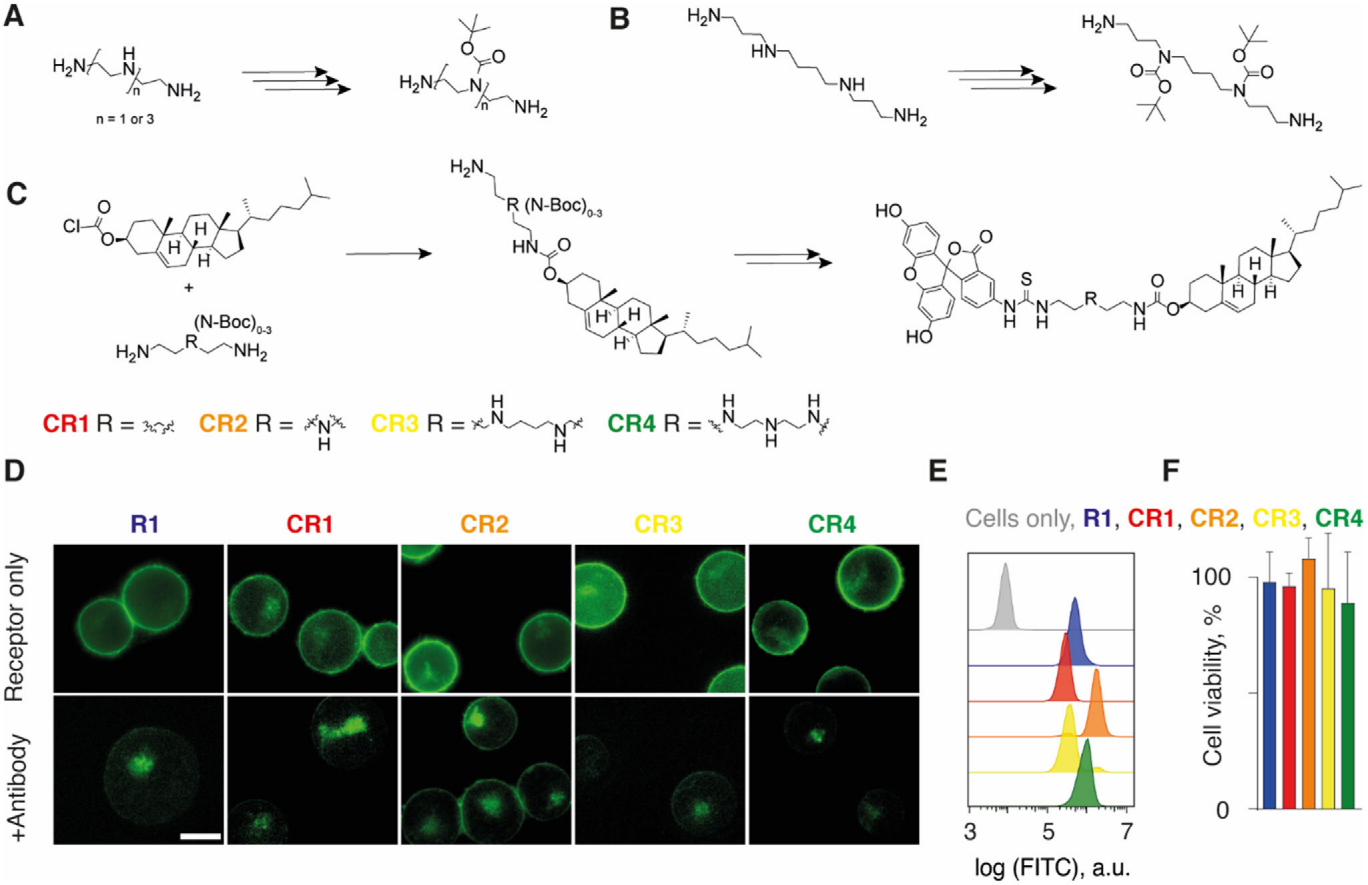
Schematic illustration of synthesis of the artificial internalizing receptors via A,B) the in situ three‐step / one‐pot protection‐protection‐deprotection reaction, C) thereafter the reaction with cholesterylchloroformate, then with fluorescein isothiocyanate, and finally the deprotection of the secondary amine groups (where present). For details on the synthesis, see the supporting information. D) Fluorescence microscopy images of MOLT‐4 cells with the artificial receptors **R1** and **CR1‐4**, with or without the addition of the cognate anti‐fluorescein antibody that quenches the fluorescence of the surface‐bound receptors. Scale bar = 10 µm. E) Flow cytometry data of the FITC fluorescence upon incorporation of the artificial receptors; F) Cytotoxicity of 10 µM artificial receptors in MOLT‐4 cells over 72 h. The presented results in F are based on three independent experiments and are shown as mean ± SD.

The artificial internalizing receptors designed in this work are structurally closer to gangliosides than to transmembrane proteins, as they contain an exofacial ligand for protein capture and a lipid anchor for association with the outer leaflet of the lipid bilayer (Figure 1). In the past, similar molecules have been designed by the Peterson group^[^ ^18^, ^19^, ^20^, ^21^ ^]^ and later used in our own work for drug delivery targeted to the artificial receptor‐engineered cells.^[^ ^22^, ^23^ ^]^ It is the high efficacy and outstanding potency of drug delivery that we observed in our prior work^[^ ^22^, ^23^ ^]^ that suggested to us that artificial internalizing receptors could be developed into a tool for targeted capture and degradation of extracellular proteins. In all prior studies, cholesterylamine (shown in the structure of the receptor **R1**, Figure 1B) has been featured as the key design criterion for the performance of artificial internalizing receptors.^[^ ^19^, ^23^, ^24^, ^25^ ^]^ However, cholesterylamine is a restrictive scaffold and allows minimal, if any, structure variation for optimization. We considered that while the amine functionality is indispensable,^[^ ^19^, ^23^, ^24^ ^]^ it possibly could be “moved” from the cholesteryl scaffold to a proximal position, placed within the linker between cholesterol and the exofacial ligand (Figure 1C). In such a case, cholesterol chemistry can be performed using the readily available cholesterylcarbamates and carbonates. Toward this end, we developed a synthetic path to employ polyamines as the linker between cholesterol and the recognition ligand. The synthesis involved a three‐step/one‐pot protection‐protection‐deprotection reaction cascade^[^ ^26^, ^27^ ^]^ that afforded homo‐bifunctional linkers with a primary amine on either end (**Figure** **2**A,B). Of these, one amine was functionalized using the commercially available cholesterylchloroformate. Following purification, the second amine was reacted with fluorescein‐5‐isothiocyanate (FITC). Deprotection of the secondary amine(s) afforded the desired amphiphiles (Figure 2C). In total, we have synthesized four new candidate artificial receptor molecules, with the number of amine groups varied from zero to three (denoted as **CR1** through **CR4**, respectively).

Chemical engineering of mammalian cells using the newly synthesized receptor molecules was first conducted using MOLT‐4 cells (T lymphoblast cell line). In these experiments, for benchmarking, we also used the receptor molecule from our previous studies,^[^ ^22^, ^23^ ^]^ which features cholesterylamine as the artificial internalization anchor (Figure 1B, denoted as **R1**). Receptor installation into MOLT‐4 cells was performed using a solution‐based protocol whereby receptor molecules, from concentrated solutions in dimethylsulfoxide (DMSO), were administered directly to the complete cell media containing the cells. The final concentration of DMSO in the media was kept at 0.2%, which is significantly lower than e.g., the DMSO content in the suspensions of chimeric antigen receptor T cells (CAR T) approved for use in humans.^[^ ^28^ ^]^ Following a 2‐h incubation, the cells were washed twice and thereafter cultured in fresh complete growth medium. Receptor installation into cells was visualized using fluorescence microscopy (Figure 2D). For all candidate receptor molecules, this protocol afforded cells with a strong fluorescence at the wavelength corresponding to the emission of light by the exofacial ligand, fluorescein. The addition of the cognate monoclonal anti‐fluorescein antibody significantly decreased the intensity of fluorescence at the cell surface, due to the fluorescence quenching upon antibody binding to the fluorophore.^[^ ^29^ ^]^ These results validate that the simple solution‐based protocol afforded rapid installation of the cholesterol‐containing molecules into the plasma membrane of the cells. Quantitatively, cell fluorescence was comparable for all candidate receptor molecules, as measured using flow cytometry (Figure 2E). This result indicates that the cholesterylamine receptor **R1** and cholesterylcarbamates **CR1‐CR4** are similarly suited to incorporate into the plasma membrane. We used fluorescence quenching of the receptor by the cognate antibody to quantify the cell surface‐resident receptors relative to the total pool of receptor molecules associated with the cell.^[^ ^22^ ^]^ These experiments revealed that for at least 24 h of cell culture, a significant fraction of fluorescein was accessible to the added antibody and thus should be available for drug delivery applications and for capturing the extracellular protein (Figure S2, Supporting Information). We also quantified the cytotoxicity of receptors at concentrations up to 10 µm, over 72 h of cell culture, and observed that cell viability remained unaltered (Presto Blue cell viability assay, Figure 2F).

Next, we performed a dose‐response study using receptor‐engineered MOLT‐4 cells and the cognate antibody drug conjugate (ADC).^[^ ^23^ ^]^ The latter contained monomethyl auristatin F (MMAF) as a drug, conjugated to the immunoglobulin via thiol‐to‐maleimide coupling, and equipped with a cathepsin‐sensitive (valine‐citrulline) dipeptide linker.^[^ ^23^ ^]^ By design, this ADC releases the drug upon cell entry, via lysosomal processing by the cathepsin B proteases.^[^ ^30^ ^]^ At concentrations up to 100 nm and in the receptor‐negative cells, ADC exhibited minimal if any cytotoxicity on its own. In stark contrast, the chemically engineered cells were highly susceptible to the ADC treatment in this concentration range (Figure S2, Supporting Information). This means that the five receptor molecules embedded into the plasma membrane of MOLT‐4 cells successfully mediated cell entry for the ADC and navigated the protein to lysosomal degradation. This conclusion is highly important for the use of artificial receptors in targeted protein degradation.

**Figure 3:**
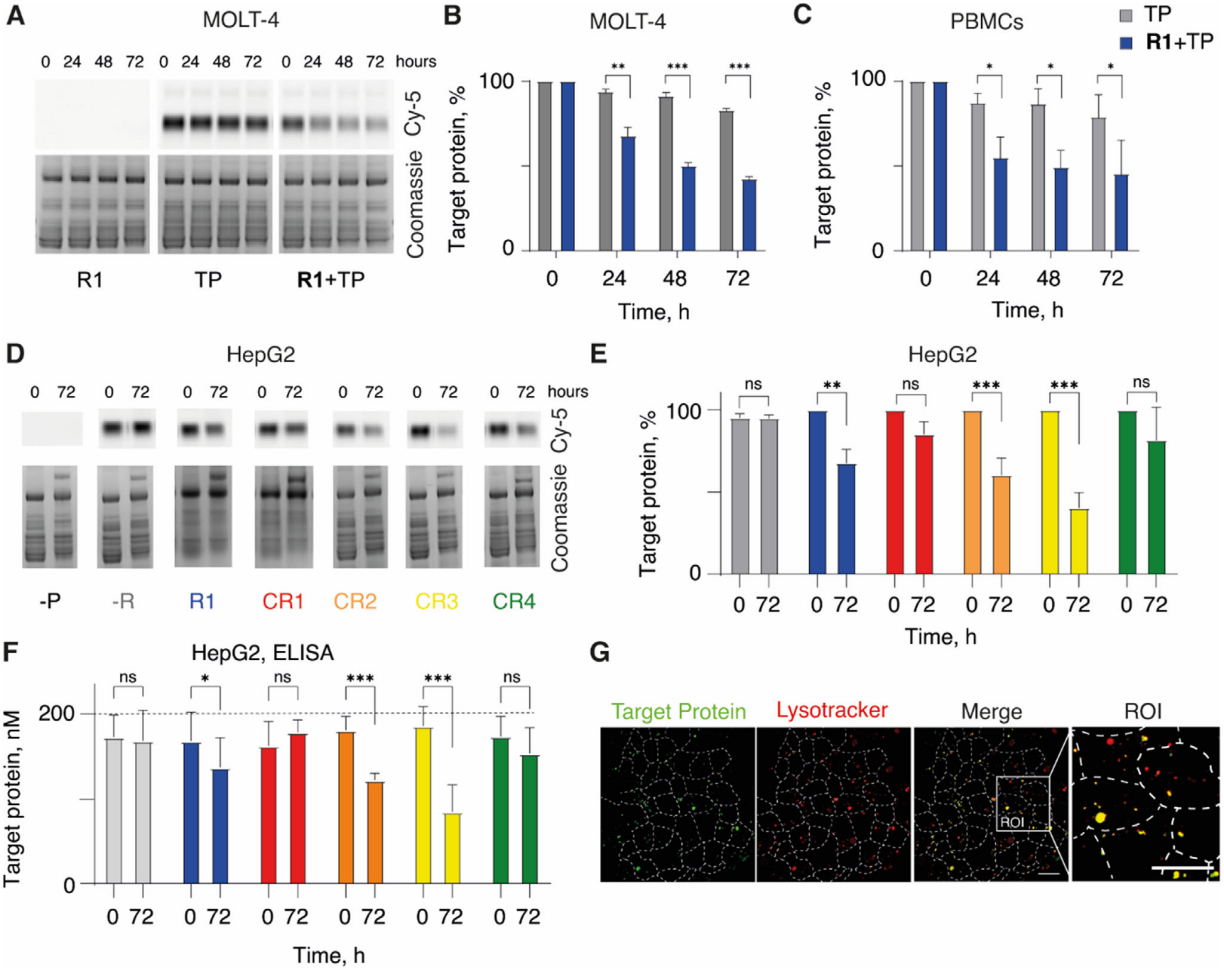
A) Representative digital photography images and B,C) pixel intensity data derived therefrom that illustrate protein capture and elimination from cell culture media by the artificial internalizing receptor **R1** in MOLT‐4 cells (A,B) and in PBMCs (C); D–F) Targeted protein capture mediated by artificial receptors in HepG2 cells whereby the residual protein content is visualized (D) and quantified (E) via gel electrophoresis or via sandwich ELISA (F). G) Confocal microscopy images of HepG2 cells treated with 10 µm **CR3**, 200 nm cy5‐labelled anti‐fluorescein antibody and lysotracker, dashed lines indicates cells, scale bar = 20 µm, ROI = region of interest, control samples can be found in supporting information. For panels A,D: full gel images including the molar mass ladder are shown in Figures S3,S4 (Supporting Information). Results in (B,C,E,F) are represented as mean ± SD of three independent experiments; statistical evaluation was performed via the two‐way ANOVA; *** = *p* < 0.001,** = *p* < 0.01, * = *p* < 0.05, ns = non‐significant. TP = target protein (anti‐fluorescein antibody).

To quantify protein capture and elimination using artificial receptors, we used the fluorescently labelled antibody (antibody = target protein, TP). This was administered to cells at a final concentration of 200 nm. Over time, the culture media was periodically sampled, and the collected fractions were analyzed via gel electrophoresis, first with fluorescence imaging and subsequently via staining for total protein content using coomassie. We found that during cell culture, the total protein pool in the media was altered insignificantly, at least according to the visual observation of the Coomassie‐stained gels (**Figure** **3**A). Using gel band quantification, we observed that the receptor‐negative MOLT‐4 cells, receiving only the target protein (“TP”), exhibited minor depletion of the anti‐fluorescein antibody, with the total eliminated fraction of the protein not exceeding 20%. In stark contrast, the receptor‐engineered cells (**R1**+TP) afforded pronounced depletion of the cognate target protein. This effect was statistically significant at each time point (24, 48, 72 h) relative to the cells that received no receptor (Figure 3B). We then performed this experiment using patient‐derived pheripheral blood mononuclear cells (PBMCs) and observed that in this case too, the receptor‐engineered cells performed well as a tool to deplete the administered antibody from the cell culture media (Figure 3C). We have previously confirmed receptor incorporation in all sub‐populations of PBMCs upon chemical engineering of the pooled cells using **R1** and this solution‐based protocol.^[^ ^23^ ^]^ Furthermore, we documented receptor‐mediated toxicity of the cognate ADC in the receptor‐engineered primary human T cells.^[^ ^22^ ^]^ It is therefore likely that protein capture and depletion to some extent is mediated by all the cells that comprise the PBMC mixture. The results presented in Figure 3A–C present the proof‐of‐concept for using artificial internalizing receptors and chemical engineering of cells toward cell‐mediated capture and depletion of extracellular target proteins.

Next, we focused on the chemical engineering of hepatocytes. The liver is the anatomical organ specializing in metabolism; it is also the organ that has been shown to receive the highest payload of cholesterol conjugates upon their in vivo administration.^[^ ^31^ ^]^ It is therefore most likely that upon the in vivo administration of the artificial receptors designed in this work, the receptor molecules will accumulate in the liver. For in vitro characterization, we used all five receptor candidate molecules (**R1, CR1‐4**), to perform the structure‐activity analysis. Much like in the MOLT‐4 cells and PBMCs, the artificial internalizing receptors performed successfully and accomplished protein depletion in the cultured hepatocytes (Figure 3D,E), as evidenced by protein visualization and quantification using gel electrophoresis. We employed sandwich ELISA to independently quantify the anti‐fluorescein antibody content, and the conclusions between the gel analysis and ELISA agreed well (Figure 3F). Confocal laser scanning microscopy confirmed that the captured target protein was navigated to the intracellular lysosomes, the cellular sub‐compartments responsible for the degradation of the internalized proteins (Figure 3G).

We observed a strong, statistically significant structure‐activity relationship for the receptor molecules regarding their performance in the selective capture and elimination of the extracellular target proteins. Under the chosen conditions, the receptor molecule **CR1** (without an ionizable amine in its structure) and, surprisingly, receptor **CR4** afforded minor protein elimination from the extracellular solution. Thus, these receptors sustained intracellular delivery of the cognate ADC (with IC_50_ values being <10 nm, Figure S2, Supporting Information), but the total removed fraction from the 200 nm solution of the antibody remained insignificant (Figure 3). This is likely due to MMAF being a highly potent toxin, whereby few copies of the internalized ADC constitute a dose sufficient for a pronounced intracellular effect (Figure S2, Supporting Information) while being an insufficient fraction of the total pool of the administered ADC (Figure 3). In contrast, the receptors **R1**, **CR2,** and **CR3**, containing 1 or 2 ionizable amines, were efficacious in both the internalization of ADC and in protein depletion, and thus can be used for chemical engineering of hepatocytes, toward selective capture and elimination of extracellular target proteins.

**Figure 4:**
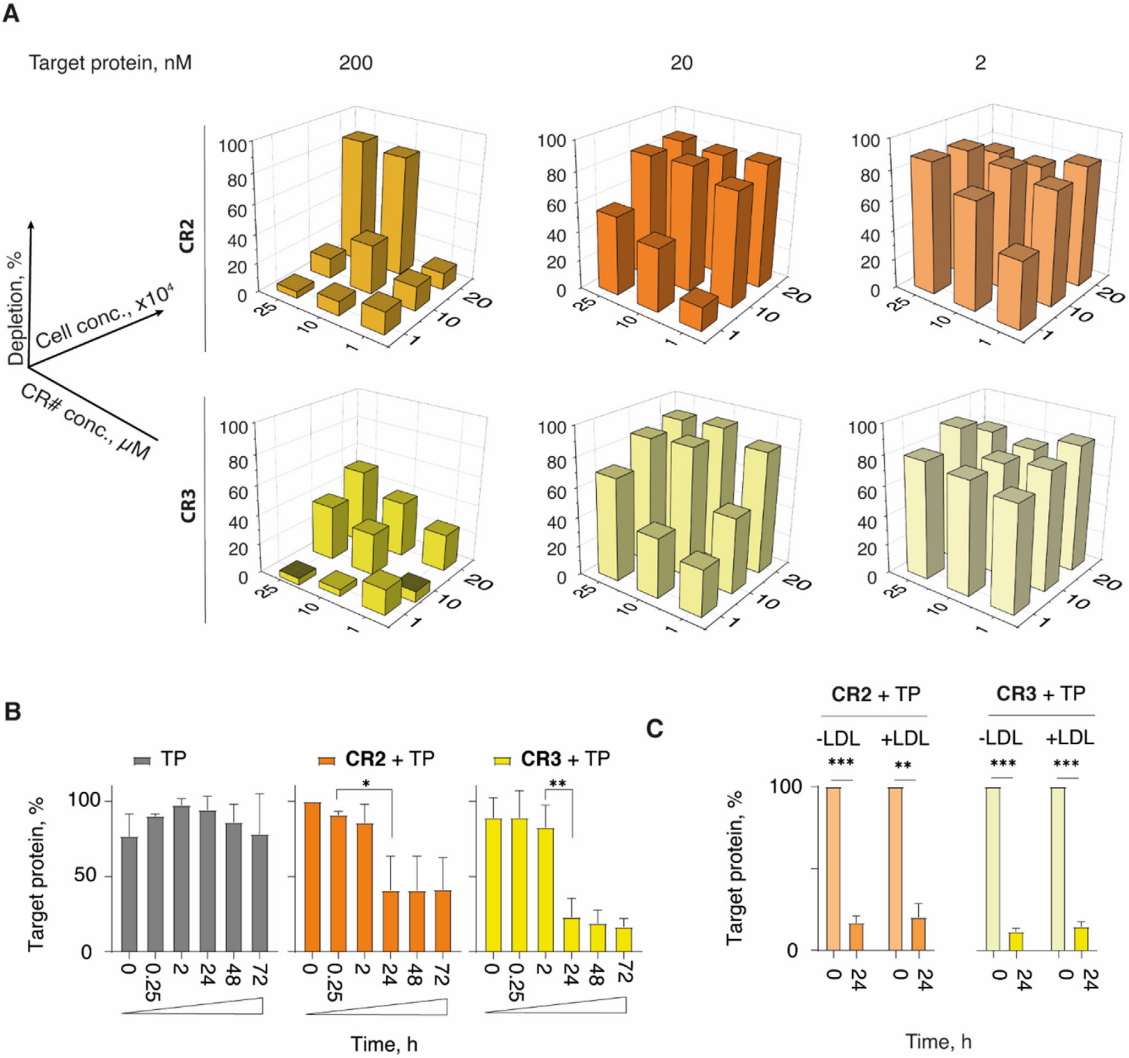
A) Screening the effect of HepG2 cell seeding density, concentration of the administered artificial receptor, and that of the target antibody on the artificial receptor‐mediated protein depletion. The experiment was carried once with two technical replicates, for screening purpose; depletion % corresponds to the removal of antibody represented as the mean of the 72 h relative to the 0 h, as quantified via ELISA. B) Residual content of the target protein in cell culture media for the HepG2 cells or their counterpart that contain **CR2** or **CR3**, over time; C) Protein depletion by the HepG2 cells with 10 µm **CR2** or **CR3** administered with or without LDL after 24 h of incubation. In panels (B,C): targeted protein content was measured via ELISA, data are represented as mean ± SD of three independent experiments; statistical evaluation was performed via two way‐ANOVA; *** *p* < 0.001, ** *p* < 0.01, * *p* < 0.05 ns = non‐significant, TP = target protein (anti‐fluorescein antibody).

The artificial receptors **CR2** and **CR3** were used to optimize the capture and elimination of the extracellular antibody. We envisioned that at least three parameters could be important to maximize the target protein capture in vitro, namely the number of cells used in the experiment, the artificial receptor feed, and the concentration of the target antibody administered to the cells. The three parameters were varied independently, and the protein content at the end of the 72 h incubation with the cells was quantified via sandwich ELISA. The results of this experiment are presented in **Figure** **4**A as the fraction of target protein removed from the solution (i.e., 100% depletion corresponds to complete removal of the target antibody). The strongest influence on the protein depletion was exerted by the concentration of the administered target antibody. At the lowest dose (2 nm), regardless of cell seeding density and the receptor feed during the cell engineering protocol, the antibody payload was exhaustively depleted using both artificial receptors. At 20 nm administered antibody, protein depletion was also very strong and with a seeding density of 10 and 20 thousand cells per well, the cells engineered with either **CR2** or **CR3** eliminated the target antibody to over the 80% depletion level. At the highest administered antibody dose of 200 nm, target protein depletion was dependent on both, cell seeding density and the receptor feed concentration and could be adapted (for the receptor **CR2**) to exhaustively deplete the 200 nm administered cognate target antibody.

During method optimization, we also considered that in vivo, cholesterol‐containing molecules are most typically adsorbed on the low‐density lipoprotein (LDL) particles found in high concentrations in the blood.^[^ ^31^ ^]^ On the one hand, the association of receptor molecules with LDL could impede their interaction with the cells. On the other, this process may change the intracellular fate of the artificial receptor molecules, as LDL is likely to interact with the natural cell‐surface LDL receptors and be internalized into the intracellular compartments. In other words, if the artificial receptors are administered with LDL, receptor molecules may start their cellular journey already within the cell, and not at the cell surface. We investigated the interaction of the receptor molecules **R1** and **CR1**‐**CR4** with LDL and found that ≈15 receptor molecules were adsorbed per LDL particle (Figure S7, Supporting Information). Furthermore, we found that the cell fluorescence was significantly higher, for all receptors except **CR2**, when the artificial receptor molecules were administered to cells in their complexes with LDL, compared to the administration of receptors without LDL. For all receptor molecules, we found that a high fraction of fluorescence could be quenched by the anti‐fluorescein antibody, which means that a significant fraction of the artificial receptor molecules was still accessible and localized at the cell surface. This fraction was very similar for the receptor molecules administered with or without LDL particles (Figure S7, Supporting Information).

For the lead compounds **CR2** and **CR3,** we conducted a time‐course study, to gain an insight into the kinetics of the target protein capture and elimination (Figure 4B). Here, we observed that the most significant changes in the protein content occur between 2 and 24 h of cell culture, which is within the range of current examples of TPD of extracellular proteins through lysosomal processing.^[^ ^6^ ^]^ These conditions were then used to quantify the antibody depletion using the cells that receive receptor molecules with or without pre‐complexing with LDL. Complexed with LDL or associating with the cells directly from the solution, the receptor molecules were well suited for target antibody capture and removal (Figure 4C).

**Figure 5:**
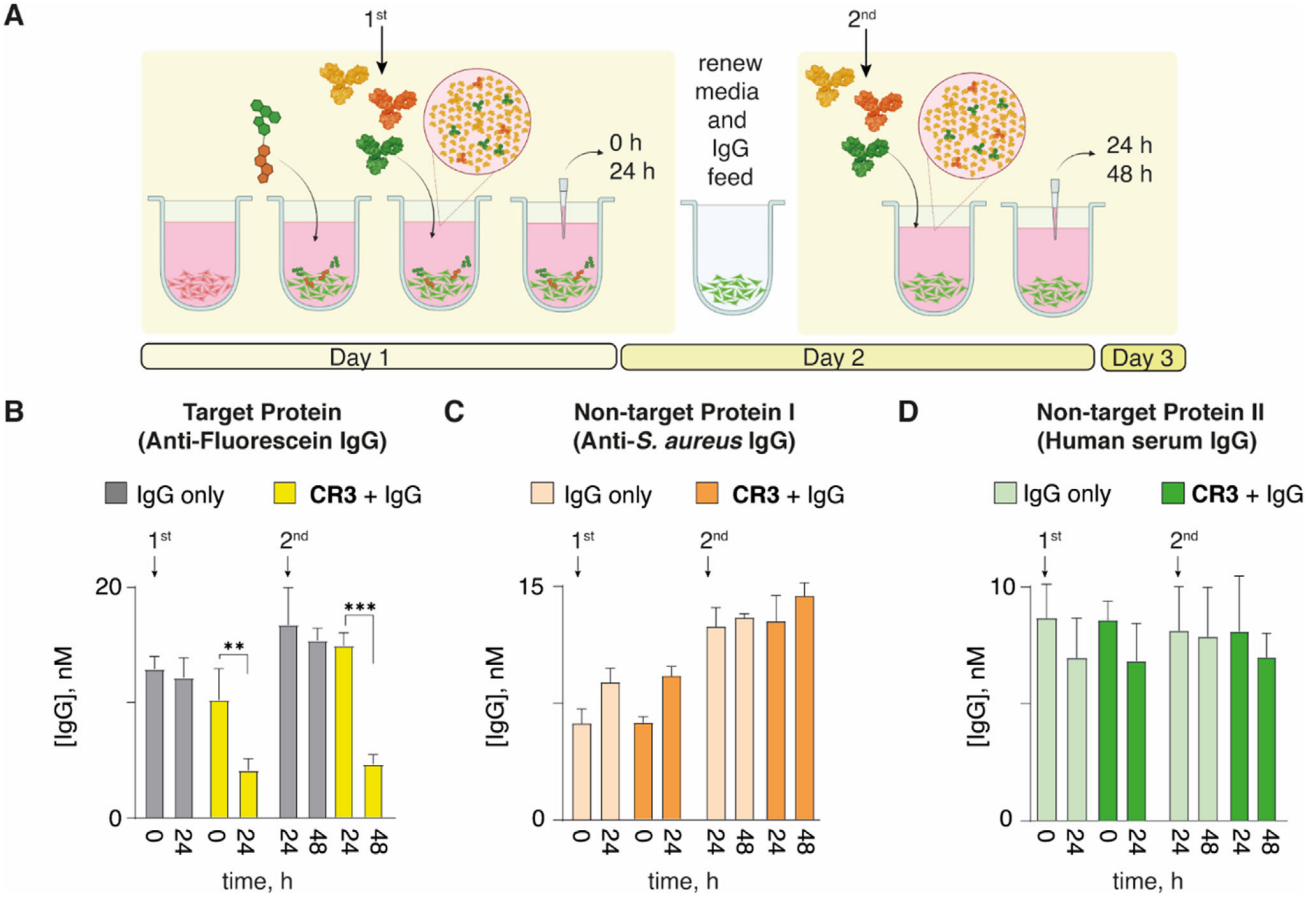
A) Schematic representation of the experiment and B) quantitative ELISA results on protein depletion using the receptor‐engineered HepG2 cells that receive 20 nm target IgG (anti‐fluorescein antibody) at 0 h and thereafter again after 24 h and non‐target proteins anti‐*S. Aureus* Rabbit IgG in C) and human serum IgG in D), quantified by Easy‐Titer or ELISA, respectively. Results in panels (B–D) are represented as mean ± SD of three independent experiments; statistical analysis is carried out using one way‐ANOVA *** *p* < 0.001, ** *p* < 0.01.

Next, we wondered if the receptor‐engineered cells could capture and eliminate the administered protein over multiple rounds of target antibody re‐addition. To test this, we focused on **CR3** and used the artificial receptor‐engineered hepatocytes to deplete the target antibody from its 20 nm solution in complete media. After 24 h of culturing, the cell media was exchanged, and cells received the second charge of the target protein in fresh media. Following an additional 24 h of culturing, the target protein content was quantified again, and we observed that the depletion of the antibody on the second round of challenge was as efficacious as the first round (**Figure** **5**A,B). This result illustrates the persistence of the artificial receptor molecules in the chemically engineered cells (which by this point in culture underwent an additional round of media change) and the continued performance of the artificial receptor molecules in capture and depletion of the target protein.

Next, we aimed to validate that the developed artificial receptors provide target specificity. To this end, hepatocytes were engineered to contain the artificial receptor **CR3** and then also incubated over time in the presence of two immunoglobulins that are not cognate to fluorescein (Figure 5C,D). The non‐target IgGs showed no sign of depletion over time. Results in Figure 5 illustrate that the developed methodology is specific to the cognate IgG, and is therefore different to the otherwise highly successful pan‐IgG degraders.^[^ ^32^ ^]^ Highly specific elimination of the cognate antibody illustrated in Figure 5 was achieved from the 20 nm solution of IgG in the complete cell culture media with a total protein content of over 90 µm (quantified via UV–vis spectroscopy). This result illustrates the envisioned performance of the artificial receptors in selectively eliminating the model disease‐causing target protein from a highly complex multicomponent environment.

The overall vision for the use of artificial internalizing receptors in vivo is that upon administration, these molecules will accumulate in the liver (and possibly in the white blood cells). Thereupon, the chemically engineered cells will continuously capture the nominated target protein, and deplete it from circulation. In doing so, the concentration of the target protein will decrease and according to the corresponding dose‐response, it should lead to decreased effects of the protein at its nominated target (e.g. a natural receptor).

**Figure 6:**
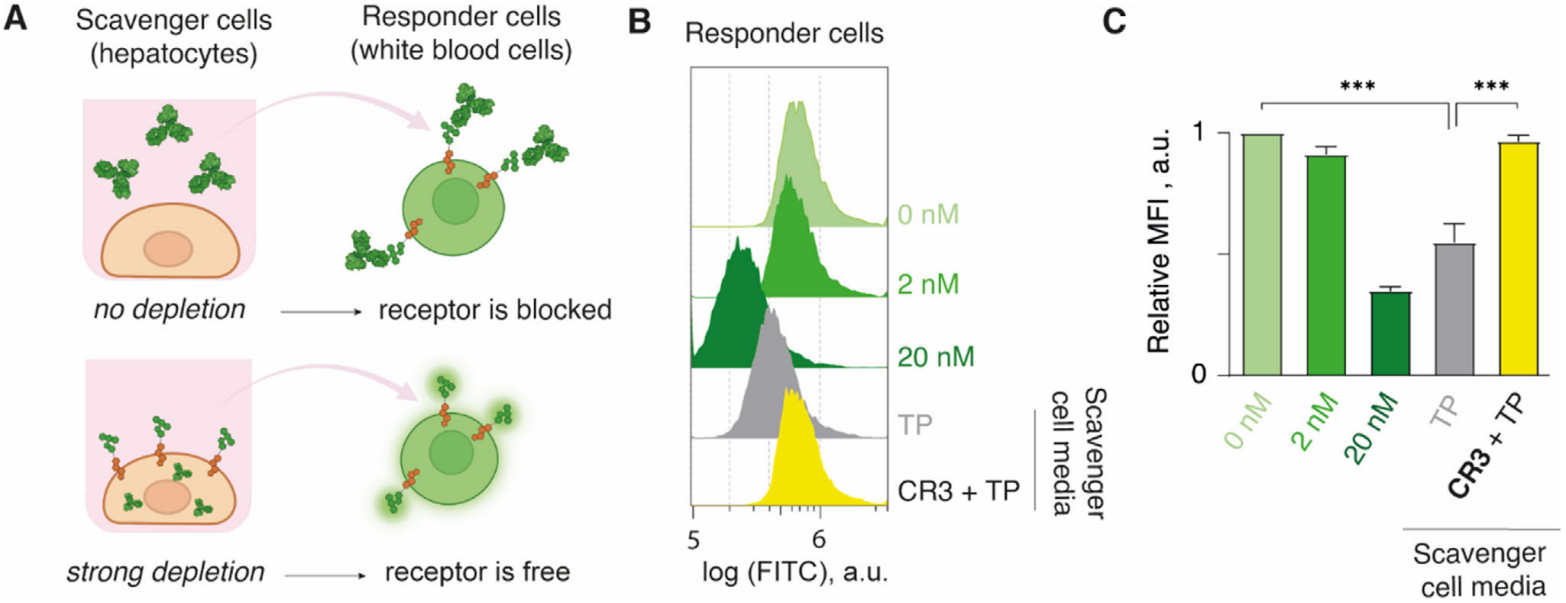
Schematic illustration A), representative flow cytometry histograms B) and quantitative flow cytometry data C) illustrating the use of HepG2 as scavenger cells, to remove the target protein and prevent its activity at the responder MOLT‐4 cells. Results in panel C are represented as mean ± SD of three independent experiments; statistical analysis is carried out using two‐way ANOVA; *** *p* < 0.001, ** *p* < 0.01. TP = target protein (anti‐fluorescein antibody).

To mimic this in vitro, we performed chemical engineering of hepatocytes as the model “scavenger” cells, using **CR3**. Separately, we performed engineering of MOLT‐4 cells as the model “responder” cells, also using **CR3** (**Figure** **6**A). Binding of the target antibody to the **CR3** on the MOLT‐4 responder cells leads to quenching of fluorescence of the latter (Figure 6B,C); this phenomenon serves as a read‐out for the association of the target protein with its cognate receptor on the responder cells. Fluorescence quenching was non‐significant when 2 nm antibody was added directly to the responder cells, but was efficacious and statistically significant at 20 nm concentration (Figure 6B,C). This concentration range is well within, or even higher than for immunoglobulins and cytokines in the human blood.

The hepatocytes were then used to capture and deplete the pool of the target antibody from the cell culture media over 24 h of incubation. The cell media from the cultured “scavenger” hepatocytes was then introduced to the “responder” MOLT‐4 cells, and the fluorescence of the responder cells was then quantified using flow cytometry (Figure 6B,C). The administration of media from the culture of hepatocytes without **CR3** (“TP”) afforded a pronounced effect of quenching of fluorescence in the “responder” MOLT‐4 cells, indicating a high concentration of the target antibody in the media. In contrast, the cell media from the hepatocytes that were chemically engineered with **CR3** (**“CR3**+TP”) afforded minor if any change in fluorescence of the MOLT‐4 cells compared to the control. This illustrates that the receptor‐engineered hepatocytes effectively captured and eliminated the target protein from the media. The engineered “scavenger” hepatocytes depleted the pool of the nominated antibody to a concentration so low that the activity of this antibody on its artificial receptor at the surface of the “responder” cell population was insignificant.

## 2 Discussion and Conclusion

In this work, we developed artificial internalizing receptors as a tool for targeted degradation of extracellular proteins. The developed artificial receptors are biorthogonal and non‐toxic, whereas the cell engineering protocol is a facile, solution‐based procedure with minimal cell handling. The same cell engineering protocol was successful for immortalized cells and donor‐derived primary human PBMCs, including both adherent and suspension cells, which has previously been highlighted as a challenge for non‐covalent engineering of mammalian cell surfaces.^[^ ^25^ ^]^ The artificial receptors, designed herein, do not have a target within the cell but empower the cell with a highly warranted, innovative function.

In the specific example illustrated herein, we were able to deplete the single nominated cognate antibody from the total pool of proteins in complete cell culture media. Removal of antibodies from circulation holds significant promise and therapies toward this goal have entered the clinic, but in their current form, these agents are pan‐IgG degraders that are unable to perform targeted action and instead deplete 40–75% of the total IgG.^[^ ^32^, ^33^ ^]^ Our approach is vastly different and we design artificial receptors to capture and degrade the one, ligand‐specific antibody rather than the total pool of immunoglobulins. By changing the extracellular binding motif from fluorescein to another, judiciously chosen ligand, artificial receptors can be readily programmed to recognize and eliminate disease‐associated proteins, within and beyond the realm of antibodies, which is the subject of our ongoing research.

Cholesterylamine exhibits outstanding persistence at the cell surface, as previously shown by the Peterson^[^ ^18^ ^]^ and the Bertozzi^[^ ^24^ ^]^ groups. In our own previous work, with application in drug delivery, we showed that the receptor **R1** remained “targetable” over at last 72 h using the cognate ADC,^[^ ^23^ ^]^ whereas the data in this manuscript illustrate that protein capture and degradation using the artificial receptors **CR2** and **CR3** is maintained over at least 48 h with continued target protein renewal. This notion highlights that the chemically engineered cellular phenotype is maintained over days, for a durable, lasting targeted protein degradation activity. It also highlights that by using artificial receptors it is possible to engineer cells toward prospective protein capture, as a preventative measure.

## Conflict of Interest

The authors declare no conflict of interest.

## Supporting information



Supporting Information

## Data Availability

The data that support the findings of this study are available from the corresponding author upon reasonable request.
