# Supplementary material for: Artificial Internalizing Receptors for Targeted Degradation of Extracellular Proteins
Source: Adv Sci (Weinh). 2025 Jul 10;12(37):e09083. doi: 10.1002/advs.202509083 (PMC12499381; doi:10.1002/advs.202509083)
Supplement: Supplementary file 1 — Supporting Information [file ADVS-12-e09083-s001.docx]

Artificial internalizing receptors
for targeted degradation of extracellular proteins

Ane B. Søgaard ^1,2^, Rikke Fabech Hansson,^1^ Mikkel Høj Nielsen,^1^ Alexander N. Zelikin^1,2*^

*^1^ Department of Chemistry, Aarhus University, Aarhus C, Denmark*

*^2^ iNano Interdisciplinary Nanoscience Center, Aarhus University, Aarhus C, Denmark*

*Email:* [*zelikin@chem.au.dk*](mailto:zelikin@chem.au.dk)

**Supporting Information**

Table of Contents

[1 Supporting Figures 4](#_Toc200439684)

[1.1 Figure S1 4](#_Toc200439685)

[1.2 Figure S2 5](#_Toc200439686)

[1.3 Figure S3 6](#_Toc200439687)

[1.4 Figure S4 7](#_Toc200439688)

[1.5 Figure S5 8](#_Toc200439689)

[1.6 Figure S6 9](#_Toc200439690)

[1.7 Figure S7 10](#_Toc200439691)

[2 Materials and Methods 11](#_Toc200439692)

[2.1 Biological Methods 11](#_Toc200439693)

[2.1.1 General information 11](#_Toc200439694)

[2.1.2 General cell culture procedures 12](#_Toc200439695)

[2.1.3 Cy-5 labelling of the monoclonal Anti-fluorescein antibody target protein 15](#_Toc200439696)

[2.1.4 Selective capture of the target protein in MOLT-4 and PBMCs 15](#_Toc200439697)

[2.1.5 General protocol for selective target protein capture in HepG2 cells 15](#_Toc200439698)

[2.1.6 Investigating lysosomal co-localization using confocal microscopy 16](#_Toc200439699)

[2.1.7 Preparation of LDL-bound receptor samples 16](#_Toc200439700)

[2.1.8 Selective protein capture in the presence of LDL 17](#_Toc200439701)

[2.1.9 Selective depletion and re-addition of extracellular proteins after 24 hours 17](#_Toc200439702)

[2.1.10 Selective protein capture effect on responder cells 17](#_Toc200439703)

[2.2 Flow Cytometry Gating Strategies 18](#_Toc200439704)

[2.2.1 Figure S8: Gating strategy from MOLT-4 cells 18](#_Toc200439705)

[2.2.2 Figure S9: Gating strategy for HepG2 cells 18](#_Toc200439706)

[2.3 Chemical methods 19](#_Toc200439707)

[2.3.1 General Information 19](#_Toc200439708)

[2.4 Synthesis Protocols 20](#_Toc200439709)

[2.4.1 General BOC-deprotection protocol 20](#_Toc200439710)

[2.4.2 Synthesis pathway of CR1 20](#_Toc200439711)

[2.4.3 Synthesis pathway of CR2 24](#_Toc200439712)

[2.4.4 Synthesis pathway of CR3 28](#_Toc200439713)

[2.4.5 Synthesis pathway of CR4 33](#_Toc200439714)

[2.5 Synthesis of MMAF Drug Linker 38](#_Toc200439715)

[2.6 NMR spectra 39](#_Toc200439716)

[2.6.1 Cadaverine (2a) 39](#_Toc200439717)

[2.6.2 Compound 3a 40](#_Toc200439718)

[2.6.3 CR1 41](#_Toc200439719)

[2.6.4 Compound 2b 42](#_Toc200439720)

[2.6.5 Compound 3b 43](#_Toc200439721)

[2.6.6 Compound 4b 44](#_Toc200439722)

[2.6.7 CR2 45](#_Toc200439723)

[2.6.8 Compound 2c 46](#_Toc200439724)

[2.6.9 Compound 3c 47](#_Toc200439725)

[2.6.10 Compound 4c 48](#_Toc200439726)

[2.6.11 CR3 49](#_Toc200439727)

[2.6.12 Compound 2d 50](#_Toc200439728)

[2.6.13 Compound 3d 51](#_Toc200439729)

[2.6.14 Compound 4d 52](#_Toc200439730)

[2.6.15 CR4 53](#_Toc200439731)

[2.6.16 Compound 5 54](#_Toc200439732)

[3 References 55](#_Toc200439733)

# Supporting Figures

## Figure S1

**Figure S1.** Fluorescence Microscopy pictures of the incorporation of receptor **CR1-CR4** and **R1** in MOLT-4 cells after the addition of 10 µM of **CR1-CR4** and **R1** for 2 h at 37 ^o^C, 5% CO_2_. Control cells received equivalents amount of DMSO only. Brightness adjusted to best fit for each image. Exposure time for Fluorescein = 200 ms. Gain = 0 and scale bar = 20 µm. The white squares are ROI and represents the pictures shown in manuscript **Figure 3**.


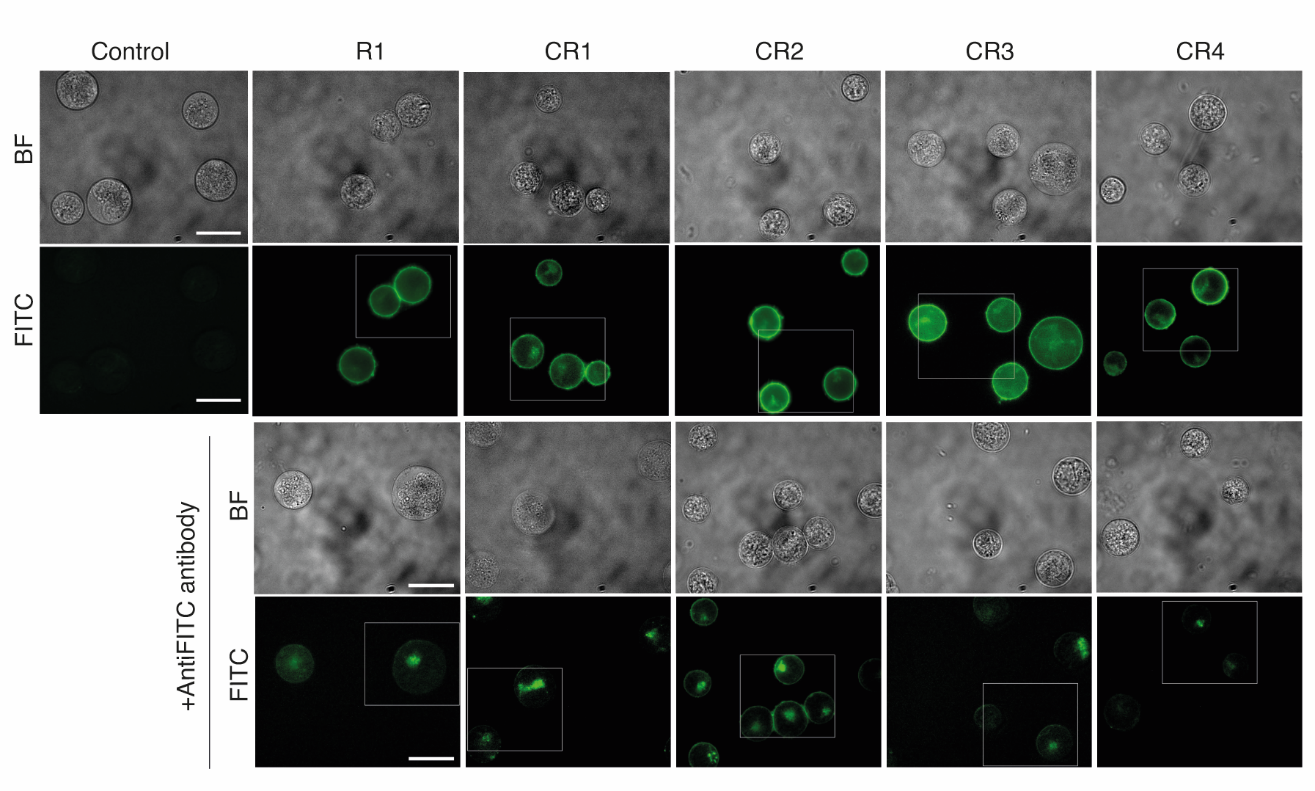


## Figure S2

**Figure S2.** **Continued** **evaluation of the new artificial carbamate receptors in human T lymphoblasts. A)** Flow cytometry data of the FITC fluorescence upon incorporation of the artificial receptors. MFI = median fluorescence intensity. **B)** Qualitative evaluation of the surface percentage of the receptors at day 1 (Day 1) right after receptor incubation or day 2 (Day 2)**,** **C)** Cytotoxicity of the artificial receptors in MOLT-4 cells over 72 hours evaluated using Presto Blue viability assay. Dose-response curves for MMAF and the corresponding ADC in MOLT-4 cells; **D)** IC50 values of the dose-response experiments presented in C; **E)** Experimental data illustrating the cell viability for cells receiving ADC at 10 or 150 nM ADC right after incubation day 1 (Day1) in (**E)** or 24 hours after incubation, day 2 (Day2) in **(F)**; The presented results are based on three independent experiments and shown as mean ± SD. Statistical evaluation was performed via a one-way or two-way ANOVA,*** p < 0.001, ** p < 0.01, * p < 0.05, ns = non-significant is not shown.


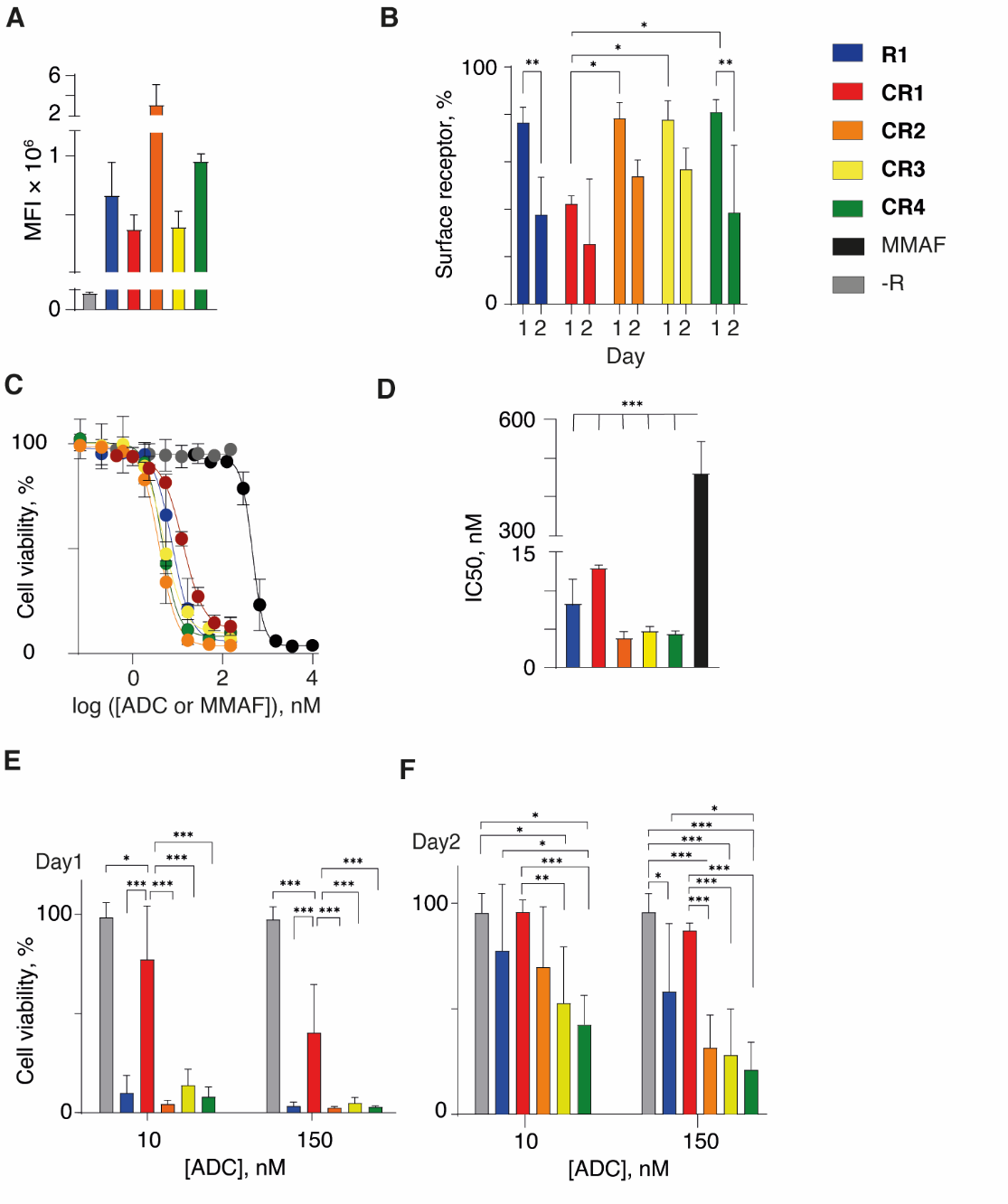


## Figure S3

**Figure S3.** Uncropped gel pictures of all three independent experiments for MOLT-4 and PBMC cell antibody capture and elimination.


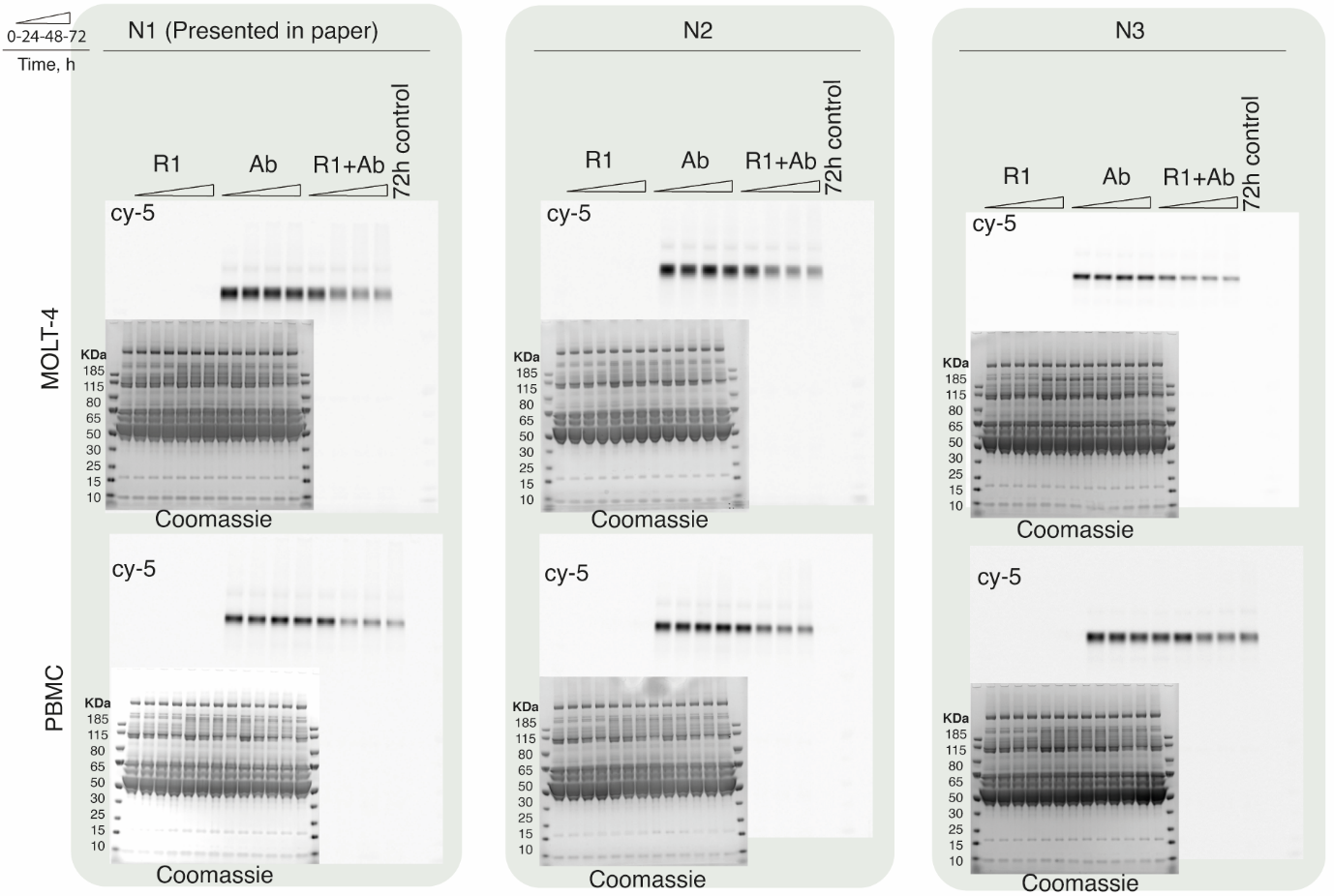


## Figure S4

**Figure S4.** Uncropped gel pictures of all three independent experiments for HepG2 antibody capture and elimination.


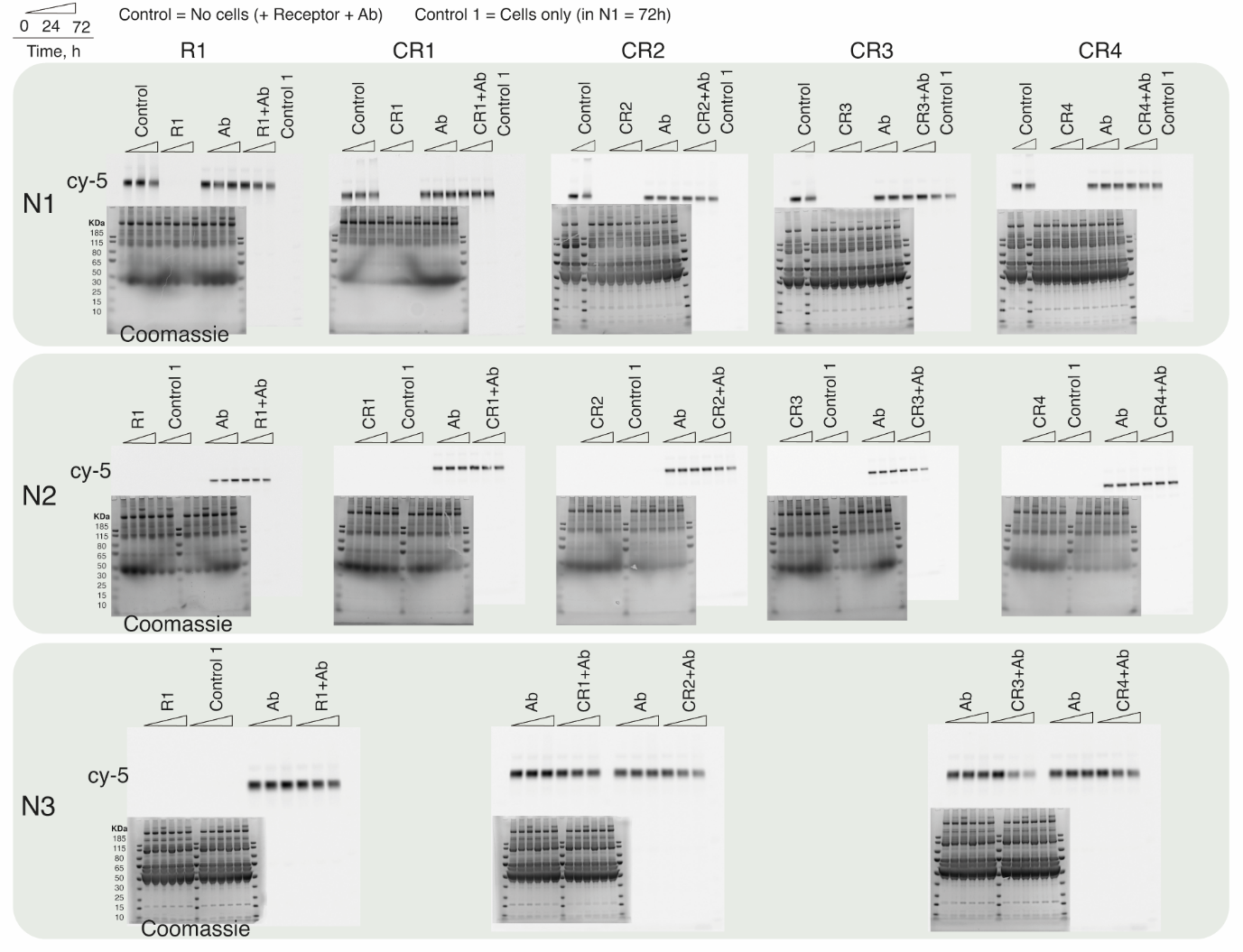


## Figure S5

**Figure S5.** Confocal microscopy images of control samples (A) and the FITC channel used for identification of cell membrane/cell area marked with dashed lines in manuscript figure 3G. Scale bar = 20 µm.


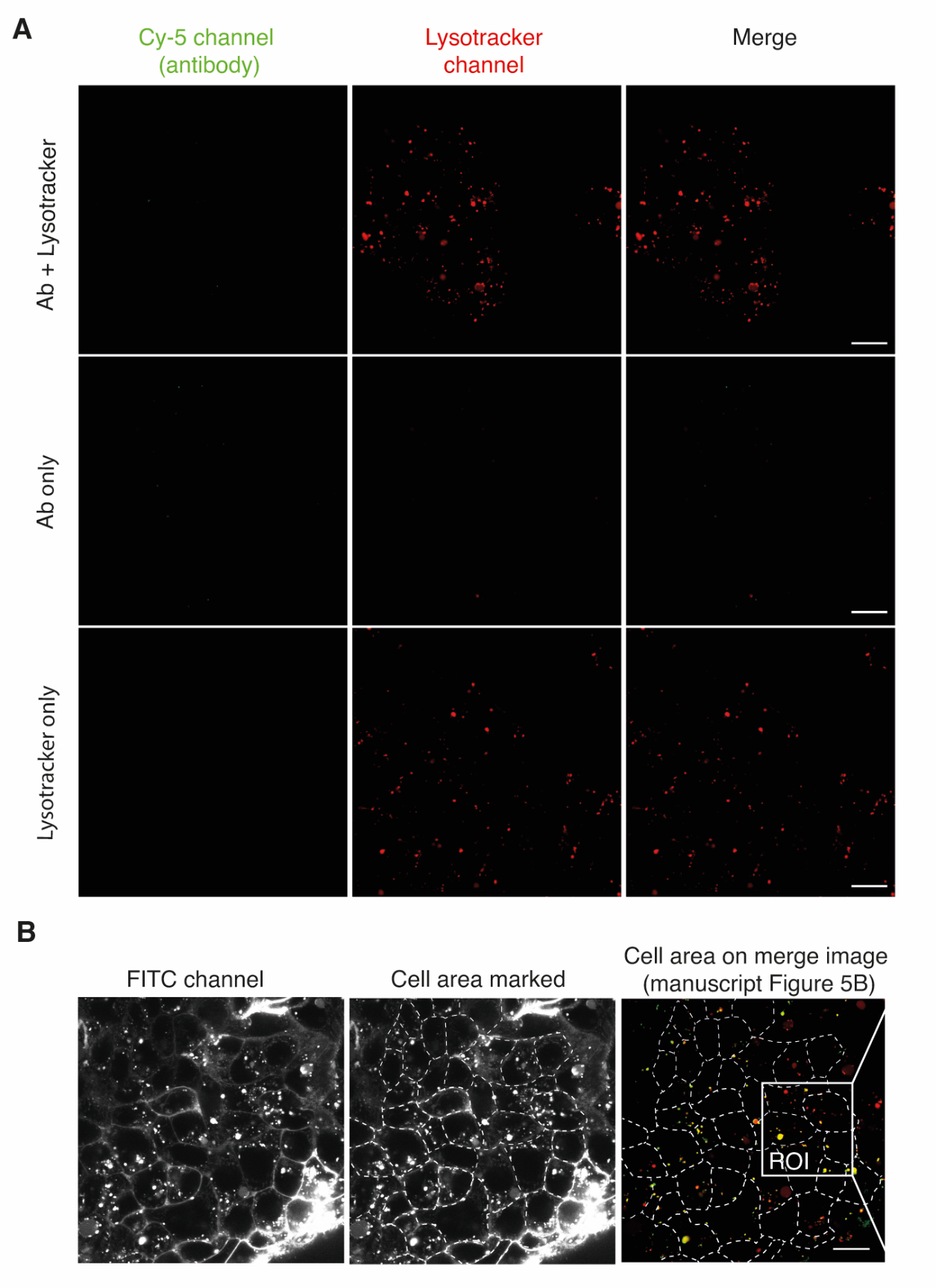


## Figure S6

**Figure S6:** Standard curve of UV-absorbance at 280 nm against LDL concentrations in M. Each sample was measured thrice, and the points were fitted against simple linear regression in GraphPad Prism 10.0.2 forcing the line to go through (0,0). The extinction coefficient was calculated based on the slope a = ε*l according to Lambert Beers law, with the path length on the Nanodrop 2000c being 0.1 cm.

## Figure S7

**Figure S7. Investigating the effect of LDL. A)** Receptor-per-LDL association ratio quantified using UV-vis absorption of LDL and fluorescence emission of the artificial receptors **R1**, **CR1-4**; **B)** Flow cytometry analysis of HepG2 cells with 10 µM **R1**, **CR1-4** administered with or without LDL, MFI = median fluorescence intensity; **C)** Qualitative evaluation of the surface percentage of the 10 µM receptors administered with or without LDL


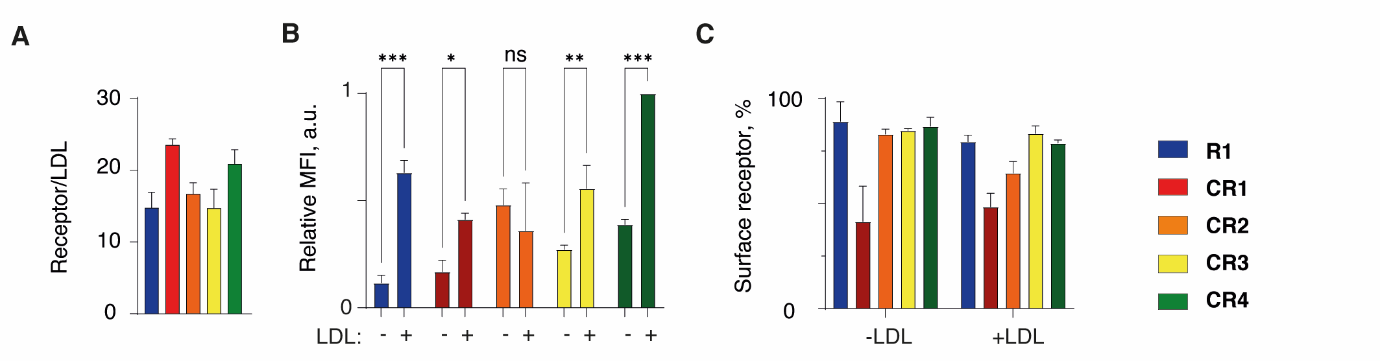


# Materials and Methods

## Biological Methods

### General information

Unless otherwise stated, all chemicals were purchased from Sigma-Aldrich/Merck and used without further purification. Anti-Fluorescein monoclonal antibody (1F8-1E4) (Cat #31242) was purchased from ThermoFisher, and the specific lot number of the used antibodies is specified within the experiment where they are used. Ultrapure water (MQ) was used from a MilliQ Direct 8 (Millipore) [18.2 MΩ • cm]. Buffy coats that were obtained from Aarhus University Hospital Blood bank and immunology. The samples were collected anonymously from volunteer donors during routine blood donation (project number p 364).

The following standard abbreviations are used throughout this section: MilliQ (MQ), Enzyme-linked immunosorbent assay (ELISA), Molecular weight cut-off (MWCO) Phosphate buffered saline (PBS), Fetal bovine serum (FBS), L-Glutamine (L-Gln), Penicillin/streptomycin (P/S), Minimum essential medium (MEM), Non-essential amino acids (NEAA), Propidium Iodide (PI), Fluorescein isothiocyanate (FITC), Bovine serum albumin (BSA), dimethyl sulfoxide (DMSO), ethylenediaminetetraacetic acid (EDTA), revolution per minute (rpm), Drug-Antibody Ratio (DAR), Antibody-Drug-Conjugate (ADC), Monomethyl auristatin F (MMAF), Lithium dodecyl sulfate (LDS), Cyanine-5 (Cy-5), Peripheral blood mononuclear cell (PBMC), Degree of labelling (DOL), Region of Interest (ROI), Target protein (TP).

**Fluorescence microscopy** was performed using a Zeiss Axio Observer Z1 equipped with a 63X lens and HXP 120C lamp. **Confocal laser scanning microscopy** was performed using a CLSM 800 inverted laser scanning confocal microscope (Zeiss, Oberkochen) with a GaAsP detector. A PlanApochromat 63x/1.4 oil DIC objective was used. See specific information on fluorophores and laser power under the experiment method description. Images were obtained using the ZEN black, Zeiss, Oberkochen Germany, and data processing was performed using Zeiss Zen 3.2, Blue edition. **Microplate reader** measurements were performed using a BioTek Synergy H1 with the Gen5 3.5 software. **Flow cytometry** was performed using a NovoCyte Quanteon 4025 flow cytometer equipped with four lasers (405 nm, 488 nm, 561 nm, and 637 nm) and 25 fluorescence detectors (Agilent, Santa Clara, CA). Data treatment was carried out using FlowJo (v.10.10.0 BD biosciences). Experimental information is stated under each specific experiment in the biological method section and the gating strategy can be found in section 2.2. **Nanodrop 2000c** (Thermo Scientific) was used to estimate the protein concentrations by measuring UV-VIS at A_280nm_. **Spin filtration** was carried out using Amicon® Ultra centrifugation columns with regenerated cellulose, the specific cutoff value is specified within the protocol of the experiments they were used. The spin filter was spun down for 10 min. at 10000 rpm and eluted by 2 min. centrifugation at 3300 rpm. **Matrix-assisted laser desorption ionization time-of-flight mass spectrometry** (MALDI-TOF MS) was performed on an Autoflex Bruker Daltonics machine in linear positive mode using the FLEX control software. The matrix consisted of 50 % acetonitrile with 0.1 % Trifluoroacetic acid and 20 g/L of Sinapic acid. Samples were prepared by mixing in a 1:1 ratio of matrix to sample (final sample conc. = 1 g/L) followed by the addition of 4 μL on an MTP anchor chip (Bruker Daltonik, Bremen, Germany). The samples were then allowed to air dry under suction for co-crystallization before the measurements were carried out. **Gel electrophoresis** was conducted with a NuPAGE® bis-tris 4-12 % gel (Thermofisher #NP0326). Samples were prepared by mixing the NuPAGE® LDS sample buffer (4X) (Thermofisher #NP0007) with 2 µg protein. Reduced samples were incubated for 5 minutes at 90 °C with 5 mM DTT. NuPAGE® MOPS SDS running buffer (20X) (Thermofisher #NP0001) was used, 150 V, 500 mA voltage was applied, and the run time was approximately 1 hour. The staining procedure was 2 times 5 min. washing in MQ followed by staining with Simply-Blue™ SafeStain (Invitrogen #LC6065) for 30 minutes. The gel was then washed with distilled water for 1 hour followed by de-staining in MQ overnight. **Gel band quantification** was performed using the Image J gel analyser tool, determining the integrated area under the curve for each band independently. This value is normalized to the time 0 h of the sample and then represented as the mean +/SD for the three independent replicates. **Digital images** were obtained using Image Quant Las 4000 equipped with a trans illuminator white light source for Coomassie stain and chemiluminescence of Cy-5 using Red Epi LED light source wavelength 630 nm. **IgG1 Mouse ELISA** was conducted according to the manufacturer's protocol (Thermofisher #88-50410-22) to quantify the amount of monoclonal Anti-Fluorescein antibody. **Human IgG (total) ELISA** was conducted according to the manufacturer's protocol (Thermofisher #BMS02091) to quantify the amount of IgG from human serum (Sigma, #I4506). **Easy-Titer^TM^ Rabbit IgG assay kit** was conducted according to the manufacturer's protocol (Thermofisher #23305) to quantify the amount of anti-*Staphylococcus aureus* polyclonal rabbit IgG (Thermofisher, #PA1-7246). **Schematics** in the figures are prepared using Biorender.com.

### General cell culture procedures

**MOLT-4**, a human T lymphoblast cell line for acute lymphoblastic leukemia, was grown as a suspension culture in RPMI-1640 Medium. **Complete growth medium** contained 10% FBS, 1% (2 mM) L-Gln, and 1% P/S. General preparation included heating of the complete media in a hot chamber to 37 °C before addition. The suspended cells were grown in 25 cm^2^ untreated culture flasks and stored upright in the incubator at 37 °C with 5% CO_2_. **Sub-culturing** was performed when cells were concentrated to around 1x10^6^ cells/mL and performed by spinning down cells at
300 x g in a centrifuge for five minutes, removing the old media and adding new complete media in a 1:3 or 1:4 split every second or third day, respectively. The cells used in all the experiments were sub-cultured at least four times after the cells were unfrozen and used no longer than passage 30.

**Isolation** **of human peripheral blood mononuclear cells (PBMCs)** was performed from Buffy coats. The samples were layered on top of 3/4 Ficoll-plaque PLUS (Cytiva #17144002) and centrifuged at 400 x g for 35 min. The PBMC layer was transferred to a new Falcon tube and washed twice with PBS. The PBMC pellet was then re-suspended in FBS with 10 % DMSO at a concentration of 6x10^6^ cells/mL and frozen down to -80°C using Mr. Frosty™ Freezing Container 1°C per minute and transferred to a cryotank for storage. Thawing of PBMCs was performed quickly and the PBMCs were cultured in 6-well plates in complete RPMI-1640 growth medium, stored in the incubator at 37 °C and 5 % CO_2_ for > 16 hours before they were diluted to a concentration of 5x10^5^ cells/mL and used within the next 4 days.

**HepG2**, a human hepatocytic cell line isolated from hepatocellular carcinoma, where grown as adherent cells in **complete growth medium** constituting Gibco MEM (sigma Aldrich # M2279) supplemented with 10% FBS, 1/ P/S, 2mM L-Gln and 1% NEAA. **Sub-culturing** was carried out when cells were 75% confluent using trypsinization (0.05% trypsin with EDTA) and seeded at a cell density of 1-3x10^4^ cells/cm^2^.

**Counting of cells** was carried out by the addition of a Trypan Blue staining solution (Gibco #1520061) in a 1:1 ratio followed by counting in an automated LUNA cell counter (Logos Biosystems). Cells were in the same procedure controlled for viability and only used at > 90%.

**Presto blue viability assay** was carried out by the addition of 10X PrestoBlue® viability reagents (Invitrogen #A13261) to each well directly in the complete cell media, followed by pipetting and shaking the plate gently to dissolve the reagent. The cells were then incubated for ~ 1 hour at 37 °C and 5% CO_2_ before the fluorescence of resorufin was quantified at λ_ex_/λ_em_ = 536/619 nm using the microplate reader.

**Statistical analysis.** Unless otherwise stated, all reported data are based on at least three independent experiments and shown as mean ± SD. Where appropriate, statistical analysis was carried out using GraphPad Prism via one - or two-way ANOVA. All cell viability data were prepared by averaging the three technical replicates, subtracting the background (media only) from the raw data, and normalizing it to the independent viability control. The viability was then plotted as a function of the logarithm of the concentration. The independent IC50 values were estimated by fitting them to a sigmoidal curve (four parameters, variable slope).

#### Fluorescence microscopy of receptor incorporation in MOLT-4 cells

MOLT-4 cells (4x10^5^ cells/mL) received 10 µM **CR1**-**CR4** or **R1** (from a 5 mM DMSO stock) in complete growth media, with a final DMSO content of 0.2%. Control cells received equivalent DMSO only. The cells were incubated for 2 hours at 37 °C, 5% CO_2_, before being washed twice in PBS and divided in two. Half of the sample received the monoclonal Anti-Fluorescein antibody at a final concentration of 0.5 µM in PBS with 2 % FBS, while the other half received PBS with 2% FBS only. All samples were kept on ice until imaged using a fluorescence microscope. See all controls and uncropped images marked with the ROI in **Figure S1** in section 1.

#### General protocol for incorporation in MOLT-4 cells

MOLT-4 cells (4x10^5^ cells/mL) received 10 µM **CR1**-**CR4** or **R1** (from a 5 mM stock in DMSO) dissolved in complete media with a final DMSO content of 0.2 %. Control cells received equivalent DMSO only. Cells were incubated for 2 hours at 37 ^ᵒ^C, 5% CO_2_, and washed twice in PBS before being used for further analysis.

#### Surface receptor percentage

**“Day 2” samples:** MOLT-4 cells were incubated with receptors as stated in the *general protocol for incorporation in MOLT-4 cells.* The cells were then dissolved in a complete growth medium, seeded in a 96-well plate, and incubated for 24 hours. “**Day 1” samples:** MOLT-4 cells were incubated with receptors as stated in the *general protocol for incorporation in MOLT-4 cells.* The cells were kept on ice for the rest of the preparation. Complete growth media was exchanged with PBS + 2% FBS containing 1 µg/mL PI with or without 0.5 µM Anti-fluorescein Ab (Lot no.: YA3809202). Fluorescein and PI fluorescence were monitored using a Quanteon flow cytometer and the experiment was performed three independent times. The gating strategy in **Figure S8** in section 2.2.

#### Cytotoxicity of receptors in MOLT-4 cells

MOLT-4 cells were incubated with receptors as stated in the *general protocol for incorporation in MOLT-4 cells.* The cells were then dissolved in a complete growth medium, seeded in a 96-well plate, and incubated for 72 hours before the Presto blue viability assay was performed.

#### Preparation of MMAF ADC

Monoclonal Anti-fluorescein antibody (lot. no: YA3809202, XE3601832, XD3575041) was buffer exchanged to PBS buffer (50 mM, 1 mM EDTA, pH 6) to a final protein concentration of > 3 g/L. 100 molar equivalent of TCEP (stock 10 g/L) was added to the Anti-fluorescein Antibody and the reaction mixture was incubated for 2 hours at 20 ^o^C. Subsequently, the reduced Ab was cooled to 4 °C before 50 equivalents of maleimide‑vc-MMAF (compound 5) was added to the solution. The conjugation was incubated 3 hours at 20 ^o^C, before it was filtered and concurrently buffer exchanged to PBS (50 mM, pH 7.4) using an Amicon filter (regenerated cellulose, 30 kDa MWCO). Next, the sample was purified using a NAP5 column (Sephadex G-25 DNA grade). The ADC solution was concentrated using an Amicon filter (regenerated cellulose, 30 kDa MWCO) and the concentration was determined using UV-VIS λ_max_ 280 nm. The conjugation of the drug and DAR was determined by MALDI-TOF spectrometry.

#### Receptor-Mediated cytotoxicity in MOLT-4 cells

MOLT-4 cells were incubated with receptors as stated in the *general protocol for incorporation in MOLT-4 cells,* re-suspended in complete growth media (2.2x10^5^ cells/mL), and seeded in 96-well plates. A dilution series of the MMAF-ADC or pristine MMAF (or fixed concentrations) was added to the samples in triplicates, and incubated for 72 hours at 37 ^o^C, 5 % CO_2_ before a Presto blue viability assay was carried out. The experiments were reproduced three independent times with technical triplicates.

### Cy-5 labelling of the monoclonal Anti-fluorescein antibody target protein

Monoclonal Anti-fluorescein antibody *(*Sigma, ref.: A16068, LOT: 70-118-121719*)* was buffer exchanged to a 0.1 M Sodium Bicarbonate buffer pH 8.3 using an Amicon® spin filter (10kDa MWCO). The antibody concentration was determined using UV-vis (A_280_). Cyanine 5 (Cy-5) NHS Ester *(*Thermofisher, ref.: A37574*)* was dissolved in DMSO to 5 g/L and added to the antibody solution in an excess of 10 molar equivalents. The reaction was left at RT for 1 hour with gentle shaking (800 rpm). The Cy-5 labeled antibody was purified and the buffer was exchanged to a 50 mM PBS pH 7.4 using an Amicon® Ultra centrifugation filter (Regenerated Cellulose, 10kDa MWCO). The final protein concentration and the DOL were determined with UV-vis (A_280_ and A_491_) measurements. The DOL for the Cy-5 Anti-fluorescein antibody was estimated to be 1.5 Cy-5/Antibody.[[1]](#footnote-1)

### Selective capture of the target protein in MOLT-4 and PBMCs

MOLT-4 cells or purified PBMCS were counted, diluted to 4x10^5^ cells/mL, and seeded in a 96-well plate with 40.000 cells/well. 10 µM **R1** was added to the +R1 samples from a DMSO stock, and an equivalent content of pure DMSO was added to the no-receptor samples. Subsequently, 0.03 g/L (200 nM) of the Cy-5 labeled monoclonal Anti-fluorescein antibody (target protein) was added from a 1.3 g/L stock in PBS pH 7.4 to the +TP samples, constituting the following samples: +R1, +TP, +R1+TP. At times 0, 24, 48, and 72 hours after the Anti-fluorescein antibody addition, the cells were re-suspended in the wells, spun down and 15 µL of the supernatant was taken from each sample and stored at 4 °C. After 72 hours all samples were collected and gel electrophoresis was conducted. Cy-5 fluorescence and Coomassie were imaged and the Cy-5 band intensity was quantified using Image J version 2.9.0/1.53 gel analyzer. See all three SDS gel replicates in **Figure S3** in section 1.

### General protocol for selective target protein capture in HepG2 cells

HepG2 cells were seeded in a 96-well plate at the 10^4^ cells/well (unless otherwise stated), and cultivated for 72 hours at 37 ᵒC and 5% CO_2_. Complete media was renewed including 10 µM artificial receptor (final DMSO % = 0.2), control cells received equivalent DMSO only. The samples were incubated for 2 hours at 37 ᵒC and 5% CO_2_ and washed twice in PBS. Cy-5 labelled target protein (Mouse IgG1 monoclonal anti-fluorescein antibody) was added to a final concentration of 200 nM, 0.03 g/L (unless otherwise stated). All samples were mixed well and from the media, samples were taken out and different time points. The target protein content was monitored using gel electrophoresis or ELISA. See all three independent SDS gel replicates in **Figure S4** in section 1.

### Investigating lysosomal co-localization using confocal microscopy

HepG2 cells were seeded on an IBIDI µ-slide 8 well ^High^ at a concentration of 30.000 cells/well and cultured for 72 h hours, before receiving 10 µM **CR3** in complete media for 2 hours at 37 °C and 5 % CO_2_, control cells received media with equivalent DMSO content (0.2 %). All samples were washed twice in PBS and incubated with or without 200 nM of the cy-5 labelled anti-fluorescein antibody potentially in combination with 75 nM Blue DND-22 Lysotracker (Invitrogen, L7525) for 2 hours at 37 °C and 5 % CO_2_. All samples were then washed three times in PBS and dissolved in live cell imaging solution (Invitrogen, A59688DJ) supplemented with 1:100 dilution of Prolong^TM^ Live Antifade Reagent (Invitrogen, P36975) and imaged at the confocal microscope. Lysotrackerblue-DND-22 was excited using the 405 nm diode laser line at 2.0 % intensity and detected at 400 nm to 640 nm wavelengths. Cyanine5 was excited using the 640 nm diode laser at 0.05 % and detected at 650 nm to 700 nm wavelengths. See all controls and uncropped pictures in **Figure S5** in section 1.

#### LDL standard curve

Samples with varying concentrations of LDL (Sigma Aldrich, LP2-2MG, 4.96 g/L stock) in PBS were prepared and the absorbance at 280 nm was measured using UV-Vis. All samples were measured thrice, and the average was plotted against the known LDL concentration. The extinction coefficient was found from the trendline (a = ε*l) by fitting with simple linear regression in GraphPad Prism 10.0.2, See supplementary **Figure S6** in section 1.

### Preparation of LDL-bound receptor samples

**CR1**-**CR4** and **R1** (from DMSO stock, 5 mM) were mixed with LDL (Sigma Aldrich, LP2-2MG, 4.96 g/L stock, lot no.: 4025970 or 4055454) to a final concentration of 382 μM receptor and 4.6 mg/mL LDL. For the control, the receptors were mixed with PBS. The samples were incubated for 2 h at 37 ^o^C, before the samples containing LDL were purified by CentriPure MINI spin Desalt Z‑25 columns. The receptor/LDL ratio was determined before and after purification by UV-VIS at 280 nm and 490 nm. If the samples were prepared in advance, the purified samples were stored at 4 ⁰C in the dark until administration onto cells.

#### Administration of receptors with LDL after purification

HepG2 cells were seeded in a 96-well plate (25.000 cells/well or 5.000 cells/well) and incubated for 24 or 72 h, respectively. The media was exchanged with media containing **CR1**-**CR4** and **R1** with and without LDL (final concentration was estimated based on UV-VIS at 280 nm and 490 nm before and after SEC purification. To control cells were added DMSO in PBS (final DMSO concentration approx. 0.2 %). The cells were incubated for 2 hours at 37 ⁰C and 5% CO_2_ before they were washed (2x PBS) and detached with trypsin (0.025 %, 25-30 μL for 5 min at 37 ⁰C, 5% CO_2_). For the rest of the preparation, the cells were kept at 4 °C. The media was exchanged with cold PBS with 2% FBS containing 0.5 μM AntiFITC antibody and 1 μg/mL PI stain. The cells were incubated on ice for 30 min before being measured on the Quanteon flow cytometer. See the gating strategy in **Figure S9** in section 2.2.

|  |  | **CR1** | **CR2** | **CR3** | **CR4** | **R1** |
| --- | --- | --- | --- | --- | --- | --- |
| Conc. [μM] | N = 1 | 9.9 | 7.1 | 8.0 | 6.1 | 9.9 |
|  | N = 2 | 8.3 | 5.2 | 8.9 | 6.7 | 4.3 |
|  | N = 3 |  |  |  |  |  |
|  | Av. | 9.1 | 6.2 | 8.5 | 6.4 | 7.1 |

### Selective protein capture in the presence of LDL

The experiment was carried out following the *General protocol for selective target protein capture in HepG2 cells*, with the only change being that the + LDL samples received receptor (**CR2**, **CR3**) in combination with LDL (ratio: 15 Receptor/LDL).

### Selective depletion and re-addition of extracellular proteins after 24 hours

The experiment was carried out following the *General protocol for selective target protein capture in HepG2 cells,* with a final concentration of target protein being 20 nM. In an additional experiment, the HepG2 cells received 20 nM Target protein (Anti-fluorescein antibody) and two non-target proteins: anti-*Staphylococcus aureus* polyclonal rabbit IgG (Thermofisher, PA1-7246) and IgG from human serum (Sigma, I4506) as a mixed protein mixture in complete media. At time 0 and after 24 hours of incubation, samples were taken out before the complete media was removed, and fresh media, including the IgG mixture, were re-added. The samples were analysed using ELISA or Easy-Titer assays.

### Selective protein capture effect on responder cells

The first part of the experiment was carried out following the *General protocol for selective protein capture in HepG2 cells* as stated above, using 10 µM **CR3** and a final concentration of target protein at 20 nM, and incubated for 24 hours. MOLT-4 cells were then prepared as stated in the *General protocol for incorporation in MOLT-4 cells* using 10µM **CR3**, and seeded at a final concentration of 3x10^5^ cells/well in a 96-well plate. The samples were kept cold and spun down (300 x g 5 min.) before the MOLT-4 media was removed and substituted with the cell media from the HepG2 cells (scavenger cell media) or for control purposes HepG2 cell media alone with or without the direct addition of 2, 20 nM target protein (anti-fluorescein antibody). All samples were incubated for 15 min. at 4 ᵒC, spun down and dissolved in PBS with 2% FBS and 1 µg/mL PI. Single-stained control samples were included. All samples were measured using a Quanteon flow cytometer. The experiments were carried out three independent times with three technical replicates. See the gating strategy in **Figure S8** in section 2.2.

## Flow Cytometry Gating Strategies

### Figure S8: Gating strategy from MOLT-4 cells

**Figure S8.** Flow cytometry gating strategy for MOLT-4 cells. Live cells were gated based on propidium iodide (PI) and only live cells were included in the final FITC histogram representation.


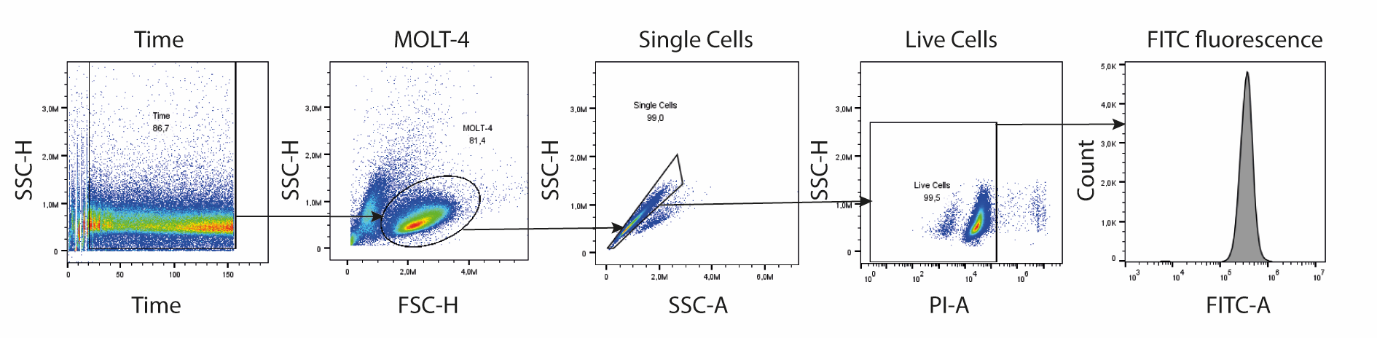


### Figure S9: Gating strategy for HepG2 cells

**Figure S9.** Gating strategy for flow cytometry analysis of HepG2 cells. Live cells were gated based on propidium iodide (PI) and only live cells were included in the final FITC histogram representation.


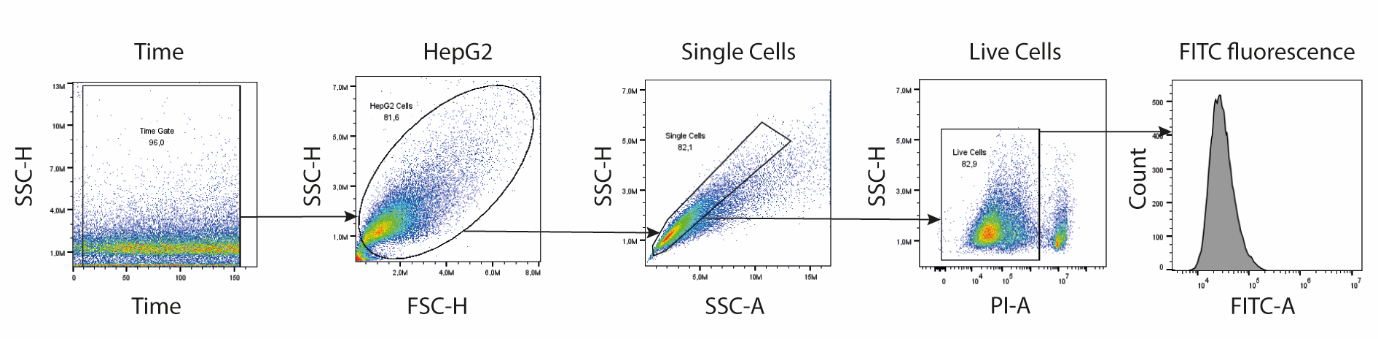


## Chemical methods

### General Information

Unless otherwise stated, all chemicals and solvents were attained from Sigma Aldrich and used without further purification. MMAF was purchased from APIChem Technology (China). *N,N*-Dimethylformamide (DMF), triethylamine (TEA), and methanol were obtained in anhydrous state. Dichloromethane was dried over aluminium oxide using an MBraun SP800 purification system. Deuterated solvents used for ^1^H-NMR and ^13^C-NMR analysis were obtained from EurisoTop. **Thin layer chromatography** (TLC) was performed using silica-coated aluminium foil plates (Merck Kieselgel 60 F254) and analyzed using UV irradiation and/or staining with KMnO_4_ or ninhydrin for visualisation. For silica column chromatography, high-purity grade silica gel (w/Ca, ~0.1%, 230-400 mesh particle size, 60Å pore size) was the stationary phase. **NMR** spectra were recorded on a Bruker AVANCE III HD spectrometer operating ^1^H-NMR at 400 MHz or ^13^C-NMR at 101 MHz. The spectra were referenced to the solvent peak, CDCl_3_ (δ_H_ 7.26 ppm, δ_C_ 77.16 ppm), CD_3_OD (δ_H_ 3.31 ppm, δ_C_ 49.00 ppm). All spectra recorded in mixtures of solvents are relative to CD_3_OD. **High-resolution mass spectrometry (HR-MS)** was performed using a Bruker Micromass LC-TOF spectrometer with electrospray ionization (ESI) and Bruker DataAnalysis were used for analysis. **High-performance liquid chromatography (HPLC)** experiments were conducted with an Agilent 1260 Infinity II connected to a ZORBAX Eclipse XDB-C8 column with particle size 5 µm, inner diameter of 4.6 mm, and length of 150 mm from Agilent (flow rate: 0.4 mL/min). The mobile phase was a combination of ultrapure water with trifluoroacetic acid (TFA, 0.1% v/v%) and HPLC grade acetonitrile with TFA (0.1% v/v%). The sample concentration was 0.5 mM receptor (**CR1-CR4**) in DMSO and 10 µL sample was injected, with UV detection at 220 nm and 254 nm. The method was 30% MeCN to 100% MeCN over 15 min, isocratic between 15 and 20 min, and 100% MeCN to 30% at 23 min**.** **Software and code.** Following software was used for data collection and/or analysis: Agilent Openlab CDS Acquisition 2.5 (HPLC); Astra 7.3.2 (SEC); Gen5 3.10 (plate reader); OriginPro 2018 (data processing and plotting); Graphpad Prism v. 9 (data processing, plotting, statistical analysis); Bruker Compass DataAnalysis 4.2 (MS data); MestReNova v14.2.0 (NMR).

## Synthesis Protocols

### General BOC-deprotection protocol

Compound **4b-4d** (1 eq.) was dissolved in CH_2_Cl_2_ (13 mM of compound **4b-4d**) before trifluoroacetic acid (100 eq.) was added dropwise. The reaction mixture was stirred in the dark at room temperature for 2-3.5 h before it was concentrated *in vacuo* and lyophilized resulting in the product as a yellow solid in quant. yield.

### Synthesis pathway of CR1

#### Synthesis of Cadaverine (2a)

**2a Cadaverine**

**C_5_H_14_N_2_**

Pentane 1,5-diamine dihydrochloride (2004 mg, 11.42 mmol, 1 eq.) was dissolved in a solution of KOH (1684 mg, 30.02 mmol, 2.6 eq.) in MeOH (10 mL). The reaction mixture was stirred for 10 min at which the KCl salt precipitated out. The slurry was filtered and washed with CH_2_Cl_2_ before the solvent was removed *in vacuo*. The crude liquid was redissolved in CH_2_Cl_2_ and dried over MgSO_4_, filtered, and concentrated *in vacuo* resulting in deprotonated cadaverine as a yellow liquid in quantitative yield.

^1^H NMR (400 MHz, CDCl_3_) δ_H_ (ppm) 2.69 (t, 4H, *J*_CH2,CH2_ 6.9 Hz, CH_2_C*H*_2_NH_2_), 1.51‑1.40 (m, 4H, C*H*_2_CH_2_NH_2_), 1.40-1.29 (m, 2H, CH_2_C*H*_2_CH_2_), 1.28-1.12 (bs, 4H, N*H*_2_).

^13^C NMR (100 MHz, CDCl_3_) δ_C_ (ppm) 42.2 (2xCH_2_*C*H_2_NH_2_), 33.7 (2x*C*H_2_CH_2_NH_2_), 24.2 (*C*H_2_).

HRMS (ESI) Calcd. for C_5_H_14_N_2_+H^+^ m/z 103.1230, found m/z 103.1233.

#### Synthesis of compound 3a

**3a**

**C_33_H_58_N_2_O_2_**

Deprotonated cadaverine (**2a**) (0.42 mL, 3.6 mmol, 8 eq.) was dissolved in anhydrous CH_2_Cl_2_ (2 mL) and cooled to 0 ⁰C before DIPEA (0.62 mL, 3.6 mmol, 8 eq.) was added. In another flask at 0 ⁰C, cholesteryl chloroformate (210 mg, 0.468 mmol, 1 eq.) was dissolved in anhydrous CH_2_Cl_2_ (2 mL) and added dropwise to the flask containing cadaverine while stirring vigorously. The reaction mixture was stirred at rt for 3 h before diluted with H_2_O and CH_2_Cl_2_. The organic phase was separated, and the aq. phase was reextracted with CH_2_Cl_2_ (x2), before the combined organic phases were dried over Na_2_SO_4_, filtered, and concentrated. The crude compound was purified by flash column chromatography (5% MeOH, 5% NH_4_OH in CH_2_Cl_2_→5% MeOH, 5% NH_4_OH, 5% EtOH in CH_2_Cl_2_) to yield compound **3a** as a white solid (209 mg, 0.406 mmol, 87%).

*R*_f_ (10% MeOH, 5% NH_4_OH in CH_2_Cl_2_) 0.31.

^1^H NMR (400 MHz, CDCl_3_) δ_H_ (ppm) 5.35 (d, 1H, *J*_C_*_H_*_,C_*_H_*_H_ 4.2 Hz, C=C*H*), 4.69 (t, 1H, *J*_NH,CH2_ 5.6 Hz, N*H*C=O), 4.53-4.04 (m, 1H, C*H*OCO), 3.15 (dd, 2H, *J*_gem_ 13.7 Hz, C*H*_2_NH), 2.68 (t, 2H, *J*_CH2,CH2_ 7.0 Hz, NH_2_C*H*_2_), 2.34 (dd, 1H, *J*_gem_ 12.0 Hz, *J*_CH2,CH_ 3.7 Hz, C*H*H), 2.24 (dd, 1H, *J*_CH2,CH_ 2.8 Hz, CH*H*), 2.09-1.74 (m, 7H, Chol-*H*), 1.57-0.91 (m, 30H, Chol-*H*+N*H*_2_+(C*H*_2_)_3_), 0.89 (d, 3H, *J*_C_*_H_*_3,C_*_H_* 6.4 Hz, C*H*_3_), 0.84 (d, 3H, *J*_C_*_H_*_3,C_*_H_* 6.6 Hz, C*H*_3_), 0.84 (d, 3H, *J*_C_*_H_*_3,C_*_H_* 6.6 Hz, C*H*_3_), 0.66 (s, 3H, C*H*_3_).

^13^C NMR (100 MHz, CDCl_3_) δ_C_ (ppm) 156.3 (*C*=O), 139.9 (*C*=CH), 122.6 (C=*C*H), 74.2 (*C*HO), 56.8, 56.2, 50.1, 42.4, 42.1, 40.9, 39.8, 39.6, 38.7, 37.1, 36.3, 35.9, 33.4, 32.0, 32.0, 30.0, 28.3, 28.3, 28.1, 24.4, 24.1, 23.9, 22.9, 22.7, 21.1, 20.4, 19.4, 18.8, 12.0.

HRMS (ESI) Calcd. for C_33_H_58_N_2_O_2_+H^+^ m/z 515.4572, found m/z 515.4590.

#### Synthesis of CR1

**CR1**

**C_54_H_69_N_3_O_7_S**

Compound **3a** (41 mg, 80 µmol, 2.1 eq.) was dissolved in anhydrous MeOH (1 mL) followed by addition of Et_3_N (11 µL, 80 µmol, 2.0 eq.) and subsequently cooled down to 0 ^o^C. In another flask, fluorescein isothiocyanate (16 mg, 40 µmol, 1 eq.) was dissolved in 1:1 MeOH/DMF (1 mL) and added dropwise to the flask containing compound **3a**. The reaction mixture was stirred for 25 h before it was concentrated. The crude product was purified by flash column chromatography twice (1^st^: 7% MeOH→12% MeOH + 1% NH_4_OH in CH_2_Cl_2_, 3 steps; 2^nd^: 10% MeOH→50% MeOH in CH_2_Cl_2_, 5 steps) resulting in receptor **CR1**, as an orange solid (11 mg, 12 µmol, 29%).

*R*_f_ (10% MeOH in CH_2_Cl_2_) 0.49.

^1^H NMR (400 MHz, 1:1 CD_3_OD/CDCl_3_) δ_H_ (ppm) 8.23 (s, 1H, Ar*H*), 8.04 (d, 1H, Ar*H*), 7.35 (d, 1H, Ar*H*), 7.17 (d, 2H, Ar*H*), 6.93-6.74 (m, 4H, Ar*H*), 5.58-5.52 (m, 1H, C=C*H*), 4.69-4.54 (m, 1H, C*H*O), 3.88-3.77 (m, 1H, C*H*), 3.57 (s, 1H, C*H*), 3.33 (t, 2H, C*H*_2_), 2.58-2.42 (m, 2H), 2.27-1.97 (m, 5H), 1.94-1.00 (m, 44H), 0.88 (s, 3H, CH_3_).

HRMS (ESI) Calcd. for C_54_H_69_N_3_O_7_S+H^+^ m/z 904.4929, found m/z 904.4952.

##### HPLC of CR1

**Figure S8:** HPLC trace of **CR1**.

### Synthesis pathway of CR2

#### Synthesis of compound 2b

**2b**

**C_9_H_21_N_3_O_2_**

Diethylene triamine (0.52 mL, 4.9 mmol, 1 eq.) was dissolved in anhydrous MeOH (80 mL) and cooled to -78 ⁰C before ethyl trifluoroacetate (1.33 mL, 11.2 mmol, 2.3 eq.) was added over 30 min. The reaction was stirred for an additional 30 min at 0 ^o^C, before Boc-anhydride (1.656 g, 7.590 mmol, 1.5 eq.) dissolved in MeOH (5.5 mL) and added dropwise. The reaction mixture was stirred overnight slowly reaching room temperature. Subsequently, 2M aq. NaOH (21 mL) was added, and the mixture was stirred for an additional 4 h. MeOH was removed under reduced pressure and the resulting residue was dissolved in CH_2_Cl_2_ and water. The aq. phase was extracted with CH_2_Cl_2_ (x5), and the combined organic phases were dried over Na_2_SO_4_, filtered, and concentrated *in vacuo*. The crude compound was purified by flash column chromatography (short (7-8 cm) and broad stationary phase, 20% MeOH, 2% NH_4_OH in CH_2_Cl_2_ →30% MeOH, 5% NH_4_OH in CH_2_Cl_2_, 1 step) resulting in compound **2b** (756 mg, 3.72 mmol, 77%) as a white sticky solid.

*R*_f_ (30% MeOH+ 5% NH_4_OH in CH_2_Cl_2_) 0.45.

^1^H NMR (400 MHz, CD_3_OD) δ_H_ (ppm) 3.29 (t, 4H, *J*_CH2,CH2_ 7.1 Hz, C*H*_2_x2), 2.76 (t, 4H, C*H*_2_x2), 1.47 (s, 9H, C*H*_3_x3).

^13^C NMR (100 MHz, CD_3_OD) δ_C_ (ppm) 156.3 (C=O), 79.9 (*C*CH_3_), 49.8 (*C*H_2_), 39.6 (*C*H_2_), 27.3 (*C*H_3_).

HRMS (ESI) Calcd. for C_9_H_21_N_3_O_2_+Na^+^ m/z 226.1526, found m/z 226.1535.

#### Synthesis of compound 3b

**3b**

**C_37_H_65_N_3_O_4_**

Compound **2b** (143 mg, 0.703 mmol, 3.2 eq.) was dissolved in anhydrous CH_2_Cl_2_ (1 mL) before DIPEA (0.23 mL, 1.34 mmol, 6 eq.) was added and the solution was cooled to 0 ^o^C. In another flask at 0 ⁰C cholesteryl chloroformate (100 mg, 0.223 mmol, 1 eq.) was dissolved in anhydrous CH_2_Cl_2_ (1 mL) and added dropwise to the flask containing compound **2b** while stirring vigorously. The reaction mixture was stirred at rt for 2.5 h before diluted with H_2_O and CH_2_Cl_2_. The organic phase was washed with H_2_O (x2) and brine (x1), dried over Na_2_SO_4_, filtered, and concentrated. The crude compound was purified by flash column chromatography (short and broad stationary phase, 5% MeOH, 2% NH_4_OH in CH_2_Cl_2_) to yield compound **3b** (102 mg, 0.166 mmol, 75%) as a white solid.

*R*_f_ (10% MeOH+ 2% NH_4_OH in CH_2_Cl_2_) 0.47.

^1^H NMR (400 MHz, CDCl_3_) δ_H_ (ppm) 5.77 (bs, 1H, N*H*H), 5.53 (bs, 1H, NH*H*), 5.34 (d, 1H, *J*_C_*_H_*_,C_*_H_*_H_ = 1.8 Hz, C*H*=C), 4.56-4.39 (m, 1H, C*H*-O), 3.49-3.16 (m, 6H, C*H*_2_), 2.85 (bs, 2H, C*H*_2_), 2.39-2.17 (m, 2H), 2.05-1.74 (m, 6H), 1.69-0.91 (m, 32H, BOC+Chol), 0.89 (d, 3H, *J*_CH3,CH_ 6.5 Hz, C*H*_3_), 0.84 (m, 6H, C*H*_3_), 0.65 (s, 3H, C*H*_3_).[[2]](#footnote-2)

^13^C NMR (100 MHz, CDCl_3_) δ_C_ (ppm) 156.5 (*C*=Ox2), 139.9 (*C*=CH), 122.5 (C=*C*H), 80.0 (*C*HO), 79.8 (O*C*(CH_3_)_3_), 56.7, 56.2, 50.6 (*C*H_2_), 50.0, 47.7 (*C*H_2_), 42.3, 40.7 (*C*H_2_), 40.0, 39.8, 39.6, 38.6, 37.0, 36.6, 36.2, 35.8, 31.9, 31.9, 28.5 (C(*C*H_3_)_3_), 28.3, 28.2, 28.0, 24.3, 23.9, 22.9, 22.6, 21.1, 19.4, 18.8, 11.9.

HRMS (ESI) Calcd. for C_37_H_65_N_3_O_4_+H^+^ m/z 616.5048, found m/z 616.5083.

#### Synthesis of 4b

**4b**

**C_58_H_76_N_4_O_9_S**

Compound **3b** (50 mg, 81 µmol, 1 eq.) and Et_3_N (11 µL, 81 µmol, 1 eq.) was dissolved in anhydrous MeOH (0.5 mL) and cooled to 0 ^o^C. FITC (38 mg, 96 µmol, 1.2 eq.) was dissolved in 2:1 anhydrous MeOH/DMF (0.75 mL) in a separate flask and added dropwise to the cold **3b**-containing flask. The reaction mixture was stirred in the dark at room temperature for 24 h, before it was concentrated *in vacuo* and redissolved in CH_2_Cl_2_. The organic phase was washed with H_2_O and brine, dried over MgSO_4_, filtered, and concentrated. The crude product was purified by flash column chromatography (5% MeOH in CH_2_Cl_2_ → 20% MeOH in CH_2_Cl_2_, 4 steps) resulting in the product **4b** (65 mg, 65 µmol, corr. 80%) as an orange powder.

*R*_f_ (7% MeOH in CH_2_Cl_2_) 0.37.

^1^H NMR (400 MHz, CD_3_OD + CDCl_3_) δ_H_ (ppm) 8.19 (d, 1H, *J*_ortho_ 7.8 Hz, Ar-*H*), 7.80 (d, 1H, *J*_ortho_ 7.8 Hz, Ar-*H*), 7.14 (d, 1H, *J*_ortho_ 8.3 Hz, Ar-*H*), 6.73 (d, 2H, *J*_ortho_ 8.8 Hz, Ar-*H*), 6.68 (d, 2H, *J*_meta_ 2.3 Hz, Ar-*H*), 6.54 (dd, 2H, *J*_ortho_ 8.8 Hz, *J*_meta_ 2.3 Hz, Ar-*H*), 5.32 (d, 1H, *J*_CH,CH2_ 3.4 Hz, C=C*H*), 4.45-4.33 (m, 1H, C*H*O), 3.79 (m, 2H, C*H*_2_), 3.54-3.46 (m, 2H, C*H*_2_), 3.38 (t, 2H, *J*_CH2,CH2_ 5.8 Hz, C*H*_2_), 3.35 (s, 1H, N*H*), 3.26 (t, 2H, *J*_CH2,CH2_ 5.8 Hz, C*H*_2_), 2.41-2.19 (m, 2H, Chol-*H*), 2.04-1.75 (m, 5H, Chol-*H*), 1.60-0.80 (m, 45H, BOC+Chol+N*H*+O*H*), 0.66 (s, 3H, C*H*_3_).

^13^C NMR (100 MHz, CD_3_OD + CDCl_3_) δ_C_ (ppm) 170.3 (C=O), 156.6 (C=O), 153.7 (*C*=S), 140.8 (Ar*C*), 139.7 (*C*=CH), 129.5 (Ar*C*Hx3), 125.4 (Ar*C*), 122.5 (C=*C*H), 119.9 (Ar*C*), 113.9 (Ar*C*), 111.0 (Ar*C*), 102.7 (Ar*C*Hx6), 80.8 (*C*HO), 79.8 (O*C*(CH_3_)_3_), 77.4, 74.2, 56.6, 56.1, 50.0, 49.4, 42.2, 39.7, 39.4, 38.5, 36.9, 36.5, 36.1, 35.7, 31.8, 28.1 (C(*C*H_3_)_3_), 27.9, 24.2, 23.7, 22.5 , 22.3, 20.9 , 19.1, 18.5, 11.6.

HRMS (ESI) Calcd. for C_58_H_76_N_4_O_9_S+H^+^ m/z 1005.5406, found m/z 1005.5443.

* Not all aromatic C-atoms are present due to low concentration.

#### Synthesis of CR2

**CR2**

**C_53_H_68_N_4_O_7_S**

Receptor **CR2** was synthesized following the **general BOC-deprotection protocol**.

^1^H NMR (400 MHz, CD_3_OD) δ_H_ (ppm) 8.19 (d, 1H, *J*_meta_ 1.4 Hz, Ar*H*), 7.90 (dd, 1H, *J*_ortho_ 8.4 Hz, *J*_meta_ 1.4 Hz, Ar*H*), 7.22 (d, 1H, *J*_ortho_ 8.4 Hz, Ar*H*), 6.79-6.72 (m, 4H, Ar*H*), 6.62 (m, 2H, Ar*H*), 5.49 s, 1H, N*H*), 5.31 (d, 1H, *J*_CH,C_*_H_*_H_ 4.3 Hz, C*H*=C), 4.43 (tt, 1H, *J*_a,e_ 4.6 Hz, *J*_a,a_ = 11.3 Hz, C*H*O), 4.03 (t, 2H, *J*_CH2,CH2_ 5.3 Hz, C*H*_2_), 3.44 (t, 2H, *J*_CH2,CH2_ 5.2 Hz, C*H*_2_), 3.37 (t, 2H, *J*_CH2,CH2_ 5.3 Hz, C*H*_2_), 3.35 (s, 1H, N*H*), 3.23 (t, 2H, *J*_CH2,CH2_ 5.2 Hz, C*H*_2_), 2.41-2.23 (m, 2H, Chol*H*), 2.04-1.74 (m, 5H, Chol*H*), 1.63-0.77 (m, 37H, Chol*H*), 0.68 (s, 3H, C*H*_3_).

^19^F NMR (376 MHz, CD_3_OD) δ_F_ (ppm) -73.4 (C*F*_3_).

HRMS (ESI) Calcd. for C_53_H_68_N_4_O_7_S+H^+^ m/z 905.4882, found m/z 905.4904.

##### HPLC of CR2

**Figure****S9:** HPLC trace before (black) and after (red) BOC-deprotection of **CR2**.

### Synthesis pathway of CR3

#### Synthesis of compound 2c

**2c**

**C_20_H_42_N_4_O_4_**

Spermine (298 mg, 1.47 mmol, 1.0 equiv.) was dissolved in anhydr. MeOH (25 mL) and ethyl trifluoroacetate (0.39 mL, 3.28 mmol, 2.2 equiv.) was added over 0.5 h at −46 °C. The mixture was stirred under an argon atmosphere for an additional 0.5 h at 0 °C, before di-*tert*-butyl dicarbonate (1.002 g, 4.591 mmol, 3.1 equiv.) dissolved in anhydr. MeOH (1.7 mL) was added dropwise to the reaction mixture at 0 °C, followed by stirring overnight at room temperature. 2 M aq. NaOH (6.5 mL, 13 mmol, 8.8 equiv.) was added to the solution at rt and the reaction mixture was stirred for 4 h. MeOH was removed under reduced pressure and the residue was partitioned between CH_2_Cl_2_ and water. The aqueous phase was extracted with CH_2_Cl_2_ (x5), and the combined organic phases were washed with brine, dried over Na_2_SO_4_, filtered, and the solvent was evaporated under reduced pressure. The crude product was purified by flash column chromatography (15% MeOH, 1% NH_4_OH in CH_2_Cl_2_ → 50% MeOH, 3% NH_4_OH in CH_2_Cl_2_, in 3 steps) yielding *N^4^,N^9^-*bis-*tert*-butoxycarbonylspermine (**2c**) (348 mg, 0.864 mmol, 59%) as a yellow syrup.

*R*_f_ (15% MeOH in CH_2_Cl_2_, 1% NH_4_OH) 0.17.

^1^H NMR (400 MHz, CDCl_3_) δ_H_ (ppm) 3.29-3.05 (m, 8H, 2xC*H***_2_**NHC*H***_2_**), 2.65 (t, 4H, *J*_CH2,CH2_ 6.7 Hz, 2xNH_2_C*H***_2_**), 1.71-1.54 (m, 8H, 2xN*H***_2_**, 2xCH_2_C*H***_2_**CH_2_), 1.45-1.41 (m, 22 H, 2xC*H***_2_**C*H***_2_**, 2xOC(C*H***_3_**)_3_).

^13^C NMR (101 MHz, CDCl_3_)[[3]](#footnote-3) δ_C_ (ppm) 155.7 (N*C*OO), 79.4 (O*C*(CH_3_)_3_), 46.8 (broad, *C*H_2_N), 44.5, 44.0 (*C*H_2_N), 39.5, 39.2 (*C*H_2_NH_2_), 32.6, 31.8 (*C*H_2_), 28.5 (*C*H_3_), 26.1, 25.6 (*C*H_2_).

HRMS(ES) Calcd. for C_20_H_42_N_4_O_4_+H^+^ m/z 403.3279, found m/z 403.3286.

#### Synthesis of compound 3c

**3c**

**C_48_H_86_N_4_O_6_**

Compound **2c** (147 mg, 0.365 mmol, 3.2 equiv.) and DIPEA (0.17 mL, 0.98 mmol, 8.5 equiv.) were dissolved in anhydr. CH_2_Cl_2_ (0.6 mL) at 0°C. In another flask, cholesteryl chloroformate (52 mg, 0.116 mmol, 1 equiv.) was dissolved in anhydr. CH_2_Cl_2_ (0.5 mL), cooled to 0°C and added dropwise to the solution containing compound **2c**. The reaction mixture was allowed to reach rt and stirred for 2 h under an argon atmosphere, after which it was washed with water (x3) and brine. The organic phase was dried over Na_2_SO_4_, filtered, and concentrated under reduced pressure. The crude product was purified by flash column chromatography (5% MeOH, 1% NH_4_OH in CH_2_Cl_2_) yielding compound **3c** (54 mg, 0.066 mmol, 57%) as a faint yellow oil.

*R*_f_ (5% MeOH in CH_2_Cl_2_, 1% NH_4_OH) 0.21.

^1^H NMR (400 MHz, CDCl_3_) δ_H_ (ppm) 5.53 (bs, 1H, N*H*COO), 5.35 (s, 1H, C=C*H*), 4.46 (s, 1H, C*H*O), 3.34-3.00 (m, 10H), 2.89-2.62 (m, 4H), 2.39-2.18 (m, 2H), 2.03-1.89 (dt, 2H), 1.89-0.76 (m, 62H, Chol*H*, NCH_2_C*H*_2_C*H*_2_CH_2_N, 2xOC(C*H*_3_)_3_), 0.66 (s, 3H, C*H*_3_).

^13^C NMR (101 MHz, CDCl_3_)[[4]](#footnote-4) δ_C_ (ppm) 156.2 (N*C*OO), 140.0 (CH=*C*), 122.4 (*C*H=C), 79.6 (O*C*(CH_3_)_3_), 74.1 (*C*HO), 70.6, 56.7, 56.1, 50.0, 46.8 (*), 46.4 (*), 43.6 (*), 42.3, 39.7, 39.5, 38.6, 37.5 (*), 37.0, 36.6, 36.2, 35.8, 31.9, 31.9 , 28.5 (OC(*C*H_3_)_3_), 28.2, 28.2, 28.0, 25.9 (*), 24.3, 23.8, 22.8, 22.6, 21.0, 19.3, 18.7, 11.9.

HRMS(ES) Calcd. for C_48_H_86_N_4_O_6_+H^+^ m/z 815.6620, found m/z 815.6683.

Peaks marked with * are broad peaks from the spermine linker caused by rotamers of the compound.

#### Synthesis of 4c

**4c**

**C_69_H_97_N_5_O_11_S**

Compound **3c** (54 mg, 0.066 mmol, 1.0 equiv.) and Et_3_N (18 µL, 0.13 mmol, 2.0 equiv.) were dissolved in anhydr. MeOH (0.3 mL) and cooled to 0°C. In another flask, fluorescein isothiocyanate (30 mg, 0.077 mmol, 1.2 equiv.) was dissolved in anhydr. MeOH (0.3 mL) and anhydr. DMF (0.5 mL), cooled to 0°C and added dropwise to the solution with compound **3c** at 0°C. The reaction mixture was allowed to reach rt and left stirring overnight under an argon atmosphere, after which the reaction mixture was concentrated under reduced pressure. The crude product was purified by flash column chromatography (6% MeOH in CH_2_Cl_2_→50% MeOH in CH_2_Cl_2_, in 5 steps) yielding compound **4c** (21 mg, 0.017 mmol, 26%) as an orange solid.

*R*_f_ (10% MeOH in CH_2_Cl_2_) 0.56.

^1^H NMR (400 MHz, 1:1 CDCl_3_ + CD_3_OD) δ_H_ 8.06 (s, 1H, Ar*H*), 7.91-7.75 (m, 1H, Ar*H*), 7.13 (d, 1H, *J*_ortho_ 8.2 Hz, Ar*H*), 6.73 (d, 2H, *J*_ortho_ 8.6 Hz, Ar*H*), 6.67 (d, 2H, *J*_meta_ 2.3 Hz, Ar*H*), 6.52 (dd, 2H, *J*_ortho_ 8.8 Hz, *J*_meta_ 1.6 Hz, Ar*H*), 5.35-5.30 (m, 1H, C=C*H*), 4.44-4.32 (m, 1H, C*H*O), 3.61 (t, 2H, *J*_CH2,CH2_ 6.2 Hz, C*H*_2_NHCS), 3.34 (s, 1H, N*H*), 3.28-3.12 (m, 8H, COONC*H*_2_), 3.08 (t, 2H, *J*_CH2,CH2_ 6.2 Hz, COONHC*H*_2_), 2.35-2.20 (m, 2H, C=CC*H*_2_CO), 2.03-0.77 (m, 66H, Chol*H*, NCH_2_C*H*_2_C*H*_2_CH_2_N, O(C*H*_3_)_3_), 0.65 (s, 3H, C*H*_3_).

HRMS(ES) Calcd. for C_69_H_97_N_5_O_11_S+H^+^ 1204.6978, found m/z 1204.7028.

#### Synthesis of CR3

**CR3**

**C_59_H_81_N_5_O_7_S**

Receptor **CR3** was synthesized following the **general BOC-deprotection protocol**.

*R*_f_ (9:1 CH_2_Cl_2_/MeOH) 0.24.

^1^H NMR (400 MHz, CD_3_OD) δ_H_ 8.19 (d, 1H, *J*_meta_ 1.6 Hz, Ar*H*), 7.71 (dd, 1H, *J* = 7.7 Hz, *J*_meta_ 1.8 Hz, Ar*H*), 7.17 (d, 1H, *J*_ortho_ 8.3 Hz, Ar*H*), 6.69 (d, 2H, *J*_meta_ 2.3 Hz, Ar*H*), 6.67 (d, 2H, *J*_ortho_ 8.9 Hz, Ar*H*), 6.54 (dd, 2H, *J*_meta_ 2.3, *J*_ortho_ 8.7 Hz, Ar*H*), 5.35 (m, 1H, C=C*H*), 4.44-4.32 (m, 1H, C*H*O), 3.82 (t, 2H, *J*_CH2,CH2_ 6.1 Hz, C*H*_2_NHCS), 3.34 (s, 4H, N*H*), 3.24-2.97 (m, 10H, C*H*_2_), 2.31 (m, 2H, C=CC*H*_2_CO), 2.10-0.83 (m, 46H, Chol*H*, C*H*_2_), 0.70 (s, 3H, C*H*_3_).

HRMS(ES) Calcd. for C_59_H_81_N_5_O_7_S+2H^+^ m/z 502.8001, found m/z 502.7996. Calcd. for C_59_H_81_N_5_O_7_S+H^+^ m/z 1004.5930, found m/z 1004.4964.

##### HPLC of CR3

**Figure S10:** HPLC trace before (black) and after (red) BOC-deprotection of **CR3**.

### Synthesis pathway of CR4

#### Synthesis of compound 2d

**2d**

**C_23_H_47_N_5_O_6_**

Tetraethylene pentamine pentahydrochloride (2.997 g, 8.073 mmol, 1 eq.) was dissolved in a solution of KOH (2.265 mg, 16.1 mmol, 5 eq.) in MeOH (16 mL). The reaction mixture was stirred overnight at which the KCl salt precipitated out. The slurry was filtered and washed with CH_2_Cl_2_ and dried over MgSO_4_, filtered, and concentrated *in vacuo* resulting in a di-protonated product as a sticky yellow oil in quantitative yield. 501 mg (501 mg, 1,91 mmol, 1 eq.) of this compound was dissolved in anhydrous MeOH (40 mL) and cooled to -78 ⁰C before ethyl trifluoroacetate (0.72 mL, 6.1 mmol, 3.2 eq.) was added over 30 min. After stirring for an additional 30 min at 0 ⁰C BOC-anhydride (2.693 g, 12.34 mmol, 6.4 eq.) in MeOH (5.5 mL) was added dropwise and the reaction mixture was stirred overnight at room temperature. 2M aq. NaOH (10 mL) was added following 4 h of stirring at room temperature before MeOH was removed under reduced pressure. The aq. phase was extracted with CH_2_Cl_2_ (x5), and the combined organic phases were dried over Na_2_SO_4_, filtered, and concentrated. The crude compound was redissolved in 4:1 MeOH/CH_2_Cl_2_ (25 mL) before 2M aq. NaOH (15 mL) was added, and the reaction mixture was stirred overnight. MeOH was removed under reduced pressure and the resulting aq. phase was extracted with CH_2_Cl_2_ (x5). The combined organic phases were dried over Na_2_SO_4_, filtered, and concentrated. The crude product mixture was purified by flash column chromatography (10% MeOH in CH_2_Cl_2_ → 20% MeOH + 4% NH_4_OH in CH_2_Cl_2_, 3 steps) resulting in compound **2d** (411 mg, 0.839 mmol, 44%) as a colourless oil.

*R*_f_ (10% MeOH + 2% NH_4_OH in CH_2_Cl_2_) 0.51.

^1^H NMR (400 MHz, CDCl_3_) δ_H_ (ppm) 3.31-3.12 (m, 12H, NHC*H*_2_C*H*_2_NH+NH_2_CH_2_C*H*_2_), 2.76 (t, 4H, *J*_CH2,CH2_ 6.2 Hz, NH_2_C*H*_2_), 1.65 (s, 4H, N*H*_2_), 1.40 (s, 27H, C(C*H*_3_)_3_).

^13^C NMR (100 MHz, CDCl_3_) δ_C_ (ppm) 155.7 (C=Ox2), 155.3 (C=O), 79.9 (O*C*(*C*H_3_)_3_), 51.1 (*C*H_2_), 50.4 (*C*H_2_), 45.7 (*C*H_2_), 45.4 (*C*H_2_), 40.9 (*C*H_2_), 40.5 (*C*H_2_), 28.5 (C(*C*H_3_)_3_), 28.5 (C(*C*H_3_)_3_x2).

HRMS (ESI) Calcd. for C_28_H_47_N_5_O_6_+H^+^ m/z 490.3600, found m/z 490.3623.

#### Synthesis of 3d

**3d**

**C_51_H_91_N_5_O_8_**

Compound **2d** (328 mg, 0.669 mmol, 2.1 eq.) was dissolved in anhydrous CH_2_Cl_2_ (1 mL) before DIPEA (0.23 mL, 1.34 mmol, 4.1 eq.) was added and the solution was cooled to 0 ^o^C. In another flask at 0 ⁰C cholesteryl chloroformate (145 mg, 0.323 mmol, 1.0 eq.) was dissolved in anhydrous CH_2_Cl_2_ (1 mL) and added dropwise to the flask containing compound **2d** while stirring vigorously. The reaction mixture was stirred at rt for 3.5 h before diluted with H_2_O and CH_2_Cl_2_. The organic phase was washed with H_2_O (x1) and brine (x1), dried over Na_2_SO_4_, filtered, and concentrated. The crude compound was purified by flash column chromatography (short and broad stationary phase, 5% MeOH, 2% NH_4_OH in CH_2_Cl_2_) to yield the product **3d** (258 mg, 0.286 mmol, 80%) as a white solid.

*R*_f_ (5% MeOH + 2% NH_4_OH in CH_2_Cl_2_) 0.32.

^1^H NMR (400 MHz, CDCl_3_) δ_H_ (ppm) 5.34 (d, 1H, *J*_CH,C_*_H_*_H_ 3.2 Hz, C*H*=C), 4.53-4.38 (m, 1H, C*H*O), 3.41-3.15 (m, 14H, C*H*_2_), 2.92-2.77 (m, 2H, C*H*_2_), 2.37-2.17 (m, 2H, Chol*H*), 2.13-1.73 (m, 8H, Chol*H*), 1.63-0.78 (m, 60, C(C*H*_3_)_3_, Chol*H*, N*H*_2_, N*H*), 0.65 (s, 3H, C*H*_3_).

^13^C NMR (100 MHz, CDCl_3_) δ_C_ (ppm) 155.6 (*C*=Ox2), 140.0 (*C*=CH), 122.5 (C=*C*H), 80.1 (*C*HO), 77.4 (O*C*(CH_3_)_3_), 74.3 (*C*H_2_), 56.8, 56.2, 50.1, 45.7 (*C*H_2_x2), 42.4, 40.7 (*C*H_2_x2), 40.1 (*C*H_2_), 39.8, 39.6, 38.7, 37.1, 36.7, 36.3, 35.9, 32.0, 32.0, 28.6 (2xC(*C*H_3_)_3_), 28.5 (C(*C*H_3_)_3_), 28.3, 28.3, 28.1, 24.4, 23.9, 22.9, 22.7, 21.1, 19.4, 18.8, 12.0.

HRMS (ESI) Calcd. for C_51_H_91_N_5_O_8_+H^+^ m/z 902.6941, found m/z 902.6992.

#### Synthesis of 4d

**4d**

**C_72_H_102_N_6_O_13_S**

Compound **3d** (60 mg, 67 µmol, 1 eq.) and Et_3_N (9 µL, 70 µmol, 1 eq.) were dissolved in anhydrous MeOH (0.5 mL) and CH_2_Cl_2_ (0.5 mL) before cooled to 0 ^o^C. FITC (29 mg, 75 µmol, 1.1 eq.) was dissolved in 1:1 anhydrous MeOH/DMF (0.50 mL) in a separate flask and added dropwise to the cold solution containing compound **3d**. The reaction mixture was stirred in the dark at room temperature for 24 h, before it was concentrated in vacuo and redissolved in CH_2_Cl_2_. The organic phase was washed with H_2_O and brine, dried over MgSO_4_, filtered, and concentrated. The crude product was purified by flash column chromatography (5% MeOH in CH_2_Cl_2_ → 15% MeOH in CH_2_Cl_2_, 3 steps) resulting in the product (77 mg, 59 µmol, 89%) as an orange powder.

*R*_f_ (10% MeOH in CH_2_Cl_2_) 0.65.

^1^H NMR (400 MHz, CDCl_3_ + CD_3_OD) δ_H_ (ppm) 8.42 (m, 1H, Ar*H*), 8.12-7.93 (m, 1H, Ar*H*), 7.26 (d, 1H, *J*_ortho_ 7.8 Hz, Ar*H*), 7.02 (d, 2H, *J*_ortho_ 8.3 Hz, Ar*H*), 6.84 (s, 2H, Ar*H*), 6.73 (d, 2H, *J*_ortho_ 8.7 Hz, Ar*H*), 5.49-5.41 (m, 1H, C=C*H*), 4.58-4.44 (m, 1H, C*H*O), 3.91 (s, 2H, C*H*_2_), 3.68-3.30 (m, 12H, 6xC*H*_2_), 2.49-2.30 (m, 2H, Chol*H*), 2.17-1.85 (m, 6H, Chol*H*+N*H*), 1.79-0.89 (m, 66H, C(C*H*_3_)_3_, Chol*H,* N*H*), 0.78 (s, 3H, C*H*_3_).

HRMS (ESI) Calcd. for C_72_H_102_N_6_O_13_S+H^+^ m/z 1291.7299, found m/z 1291.7353. Calcd. for C_72_H_102_N_6_O_13_S+H^+^+Na^+^ m/z 657.3596, found m/z 657.3581. Calcd. for C_72_H_102_N_6_O_13_S+H^+^+K^+^ m/z 665.3465, found m/z 665.3418.

#### Synthesis of CR4

**CR4**

**C_57_H_78_N_6_O_7_S**

Receptor **CR4** was synthesized following the **general BOC-deprotection protocol**.

*R*_f_ (10% MeOH in CH_2_Cl_2_) 0.31.

^1^H NMR (400 MHz, CD_3_OD) δ_H_ (ppm) 8.36 (d, 1H, *J*_meta_ 1.8 Hz, Ar*H*), 7.87 (dd, 1H, *J*_ortho_ 8.2 Hz, *J*_meta_ 1.8 Hz, Ar*H*), 7.24 (d, 1H, *J*_ortho_ 8.2 Hz, Ar*H*), 6.98-6.81 (m, 4H, Ar*H*), 6.74 (dd, 2H, *J*_ortho_ 8.8 Hz, *J*_meta_ 2.2 Hz, Ar*H*), 5.49 (s, 1H, N*H*), 5.36 (d, 1H, *J*_CH,CH_ 4.57 Hz, C=C*H*), 4.40 (tt, 1H, *J*_a,a_ 10.7 Hz, *J*_a,e_ 4.9 Hz, C*H*O), 4.08 (t, 2H, *J*_CH2,CH2_ 5.8 Hz, C*H*_2_), 3.98 (s, 1H, N*H*), 3.55 (t, 2H, C*H*_2_), 3.51 (m, 11H, C*H*_2_+N*H*), 3.34 (s, 3H, N*H*), 3.23 (t, 2H, C*H*_2_), 2.38-2.22 (m, 2H, Chol*H*), 2.06-1.79 (m, 6H, Chol*H*), 1.66-0.91 (m, 34H, Chol*H*), 0.87 (d, 3H, *J*_CH3,CH_ 6.6 Hz, C*H*_3_), 0.87 (d, 3H, *J*_CH3,CH_ 6.6 Hz, C*H*_3_), 0.70 (s, 3H, C*H_3_*).

^19^F NMR (376 MHz, CD_3_OD) δ_C_ (ppm) -77.3 (C*F*_3_).

HRMS (ESI) Calcd. for C_57_H_78_N_6_O_7_S+H^+^ m/z 991.5726, found m/z 991.5756. Calcd. for C_57_H_78_N_6_O_7_S+2H^+^ m/z 496.2899, found m/z 496.2900.

##### HPLC of CR4

**Figure S11:** HPLC trace before (black) and after (red) BOC-deprotection of **CR4**.

## Synthesis of MMAF Drug Linker

**5**

**C_68_H_103_N_11_O_16_**

Synthesis of mal-vc-MMAF, **5**, can be found in a previously published paper ^[1]^

## NMR spectra

### Cadaverine (2a)

### Compound 3a

### CR1

### Compound 2b

### Compound 3b

### Compound 4b

### CR2

### Compound 2c

### Compound 3c

### Compound 4c

### CR3

### Compound 2d

### **Compound 3d**

### Compound 4d

### CR4

### Compound 5

# References

[1] A. B. Søgaard, F. Skovbo, A. Tvilum, R. F. Hansson, A. N. Zelikin, *Adv. Funct. Mater.* **2024,** *34*, 2400048.

1. <https://assets.thermofisher.com/TFS-Assets/LSG/brochures/TR0031-Calc-FP-ratios.pdf> [↑](#footnote-ref-1)
2. The integral shows a higher number of protons present in the cholesterol-area, which originates from H_2_O. [↑](#footnote-ref-2)
3. Rotamers exists for this compound, therefore several peaks were observed for some carbons. [↑](#footnote-ref-3)
4. Rotamers exists for this compound, therefore several peaks were observed for some carbons. [↑](#footnote-ref-4)
